# Satellite UV-Vis spectroscopy: implications for air quality trends and their driving forces in China during 2005–2017

**DOI:** 10.1038/s41377-019-0210-6

**Published:** 2019-11-13

**Authors:** Chengxin Zhang, Cheng Liu, Qihou Hu, Zhaonan Cai, Wenjing Su, Congzi Xia, Yizhi Zhu, Siwen Wang, Jianguo Liu

**Affiliations:** 1 School of Earth and Space Sciences, University of Science and Technology of China, 230026 Hefei, China; 2 Department of Precision Machinery and Precision Instrumentation, University of Science and Technology of China, 230026 Hefei, China; 3 Key Laboratory of Environmental Optics and Technology, Anhui Institute of Optics and Fine Mechanics, Chinese Academy of Sciences, 230031 Hefei, China; 4 Center for Excellence in Regional Atmospheric Environment, Institute of Urban Environment, Chinese Academy of Sciences, 361021 Xiamen, China; 5 Key Laboratory of Precision Scientific Instrumentation of Anhui Higher Education Institutes, University of Science and Technology of China, 230026 Hefei, China; 6 Key Laboratory of Middle Atmosphere and Global Environment Observation, Institute of Atmospheric Physics, Chinese Academy of Sciences, 100029 Beijing, China; 7 Multiphase Chemistry Department, Max Planck Institute for Chemistry, 55128 Mainz, Germany

**Keywords:** Atmospheric optics, Optical spectroscopy

## Abstract

Abundances of a range of air pollutants can be inferred from satellite UV-Vis spectroscopy measurements by using the unique absorption signatures of gas species. Here, we implemented several spectral fitting methods to retrieve tropospheric NO_2_, SO_2_, and HCHO from the ozone monitoring instrument (OMI), with radiative simulations providing necessary information on the interactions of scattered solar light within the atmosphere. We analyzed the spatial distribution and temporal trends of satellite-observed air pollutants over eastern China during 2005–2017, especially in heavily polluted regions. We found significant decreasing trends in NO_2_ and SO_2_ since 2011 over most regions, despite varying temporal features and turning points. In contrast, an overall increasing trend was identified for tropospheric HCHO over these regions in recent years. Furthermore, generalized additive models were implemented to understand the driving forces of air quality trends in China and assess the effectiveness of emission controls. Our results indicated that although meteorological parameters, such as wind, water vapor, solar radiation and temperature, mainly dominated the day-to-day and seasonal fluctuations in air pollutants, anthropogenic emissions played a unique role in the long-term variation in the ambient concentrations of NO_2_, SO_2_, and HCHO in the past 13 years. Generally, recent declines in NO_2_ and SO_2_ could be attributed to emission reductions due to effective air quality policies, and the opposite trends in HCHO may urge the need to control anthropogenic volatile organic compound (VOC) emissions.

## Air pollution: satellite sees significant trends in China

Satellite-based observations of air pollution in China reveal a recent declining trend in the levels of nitrogen dioxide (NO_2_) and sulfur dioxide (SO_2_), but rising levels of formaldehyde (HCHO). Chengxin Zhang and colleagues at the University of Science and Technology of China, with co-workers at other Chinese research centers, analyzed ultraviolet-visible spectroscopy data from the Ozone Monitoring Instrument of NASA’s EOS-Aura satellite. Meteorological effects such as winds, temperature and humidity caused day-to-day and seasonal fluctuations, but human activity appears responsible for long term trends. Declines in NO_2_ and SO_2_ levels occurred from 2011, across most regions, and are attributed to effective air quality policies. The rising trend in HCHO levels suggests that new measures may be needed to control the release by human activities of that and other volatile organic compounds.

## Introduction

Nitrogen dioxides (NO_2_), sulfate dioxides (SO_2_), and formaldehyde (HCHO) are short-lived and reactive trace gases that play important roles in atmospheric chemistry and air pollution^1^. NO_2_ and SO_2_ can be converted into secondary inorganic aerosols, i.e., nitrate and sulfate, respectively, via reactions with OH radicals^2^. HCHO usually originates from the photochemical reactions of volatile organic compounds (VOCs) and can be used as a proxy for the total reactivity of VOCs^3^. The sources of VOCs include fire, vegetation and anthropogenic emissions^4^. VOCs are important precursors of secondary organic aerosols and ozone (O_3_)^5^. Anthropogenic emissions from the power, industrial, residential, transportation, and agricultural sectors enhance the concentrations of these gases in the troposphere, especially in the boundary layer, over urban areas.

Spectroscopy techniques greatly advance the comprehensive understanding of air pollution evolution^6–9^, especially with the broad application of ground-based and space-based passive and active remote sensing. Since the 1990s, numerous space-borne ultraviolet-visible (UV-Vis) spectrometers, e.g., the Global Ozone Monitoring Experiment (GOME)^10^, SCanning Imaging Absorption SpectroMeter for Atmospheric CHartographY (SCIAMACHY)^11^, Ozone Monitoring Instrument (OMI)^12^, and Global Ozone Monitoring Experiment–2 (GOME-2)^13,14^, have achieved the global monitoring of atmospheric trace gases, including NO_2_, SO_2_, HCHO, and O_3_, by using their unique absorption signatures in a shorter wavelength range (250–500 nm). In principle, the numerical inversion methods of these key atmospheric variables could be achieved by incorporating radiative transfer simulations on the interactions of solar scattered light within the atmosphere^15^.

With rapid economic growth and urbanization, central and eastern China have been suffering from severe air pollution over the last decade^16–18^. Anthropogenic pollutant emissions are a primary cause of ambient air pollution. In addition, meteorological factors could also impact air quality through atmospheric processes such as formation, transport, convection and both the dry and wet deposition of air pollutants^1^. The role of emissions and meteorological conditions in the evolution of air pollution has been investigated for cases such as heavy pollution episodes in winter in Beijing^19^ and several important international events during which the government has conducted strict emission controls in Beijing and Nanjing^20–22^. Nevertheless, there still remain a series of unanswered questions, e.g., the separation of meteorological effects from the human-induced variations in air pollution and the evaluation of the effectiveness of emission control measures or air quality policies implemented by the Chinese government, such as the Air Pollution Prevention and Control Action Plan (APPCAP) issued in 2013^23^.

A number of studies have focused on the relative contributions of emissions and meteorological conditions. However, their conclusions were restricted to either small geographical areas or short periods based on limited in situ measurements. Due to the advantage of satellite observations in terms of spatiotemporal coverage, some studies have clearly captured the temporal variability in tropospheric air pollutants over China and attributed the long-term pollutant trends to the variation in anthropogenic emissions such as nitrogen oxides (NO_x_) and SO_2_^24,25^. However, to better understand the effects of anthropogenic emissions and emission control measures, the influences of meteorological conditions should be separated from the long-term satellite-observed air quality trends.

In this study, satellite spectroscopic measurements from the OMI were first used to retrieve the tropospheric abundances of NO_2_, SO_2_ and HCHO over central and eastern China (20°–45°N, 100°–125°E), and then air quality trends were analyzed based on the derived spatiotemporal data. The OMI was selected due to its high signal-to-noise ratio, fine spatial resolution, stable spectral performance, and most importantly, long temporal coverage^26^ compared to other satellite sensors of its type, such as GOME-2, SCIAMACHY, etc. Several heavily polluted and densely populated regions were focused on, e.g., Beijing-Tianjin-Hebei (BTH), Changjiang River Delta (YRD), Zhujiang River Delta (PRD), and Sichuan Basin (SCB). These regions have drawn increasing scientific attention to their widespread air pollution in the last decade. Due to the complex interactions and feedbacks between meteorological conditions and air quality^19^, separating the effects of emission variations on air quality trends from meteorological factors remains challenging. Here, we have implemented generalized additive models (GAMs) to quantitatively assess the impacts of meteorological and anthropogenic variables on air quality variations for typical megacities over these regions. The GAMs make use of penalized smoothing splines, which could address the complex non-linearity existing in air quality and meteorology research^27^. Contrary to previous studies^20–22^ relying on the atmospheric chemistry model, this novel statistical method based on long-term satellite observations provides an explicit solution for quantifying natural and anthropogenic impacts and assessing the role of emission control measures on air quality trends.

## Results

### The spatiotemporal variability in OMI-measured air pollutants

**Fig. 1:**
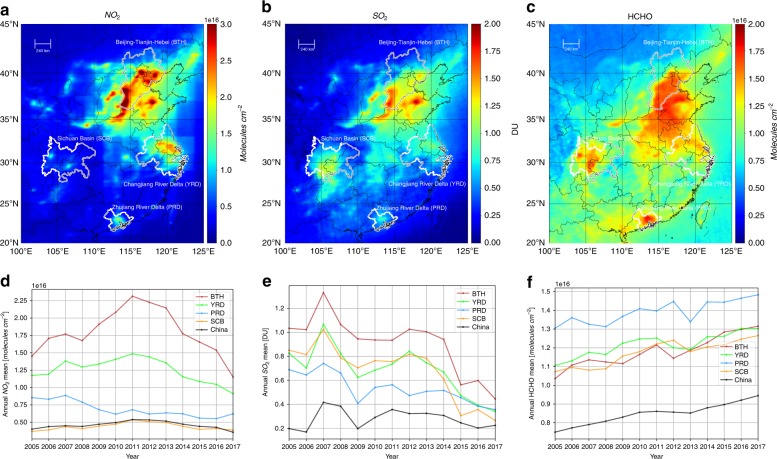
The spatiotemporal characteristics of OMI-measured pollutant concentrations. **a**–**c** Tropospheric mean VCDs during 2005–2017 for NO_2_, SO_2_, and HCHO, respectively. The regional boundaries of BTH, YRD, PRD and SCB are delineated by gray lines. **d**–**f** The corresponding annual mean VCDs for NO_2_, SO_2_, and HCHO, respectively. Note that the mean values for the four regions and China are shown by different colors

The spatial distributions of the tropospheric VCD retrievals of NO_2_, SO_2_, and HCHO during 2005–2017 are presented in Fig. 1a–c, respectively. Extremely high concentrations of air pollutants can be clearly found with a large spatial coverage over typical industrial and densely populated regions in China, e.g., BTH, YRD, PRD, and SCB. Spatially, BTH suffered from the most severe NO_2_ and SO_2_ pollution levels compared to other regions. A large hotspot of HCHO pollution can also be seen over these industrial areas, especially in PRD. Based on the VCD variation patterns shown in Fig. 1d–f, we concluded that the interannual variability in OMI-measured pollutants during 2005–2017 over central and eastern China was not monotonically increasing or decreasing but had different temporally varying regimes for individual gas species and regions. In addition, the temporal trends of these pollutants were spatially consistent for the satellite ground pixels within each region (See Fig. S1). Therefore, for each region, we could use the spatial average to analyze the regional trends in air pollution and choose one typical megacity to explore its driving forces regarding air quality trends.

For the tropospheric NO_2_ column, the increases were estimated as 59.5, 26.7, and 45.2% for the BTH, YRD and SCB regions from 2005 to 2011, followed by significant decreases of 74.1, 45.1, and 33.2% during 2012–2017, respectively. In contrast to other regions, PRD showed a continuous decrease in the NO_2_ column at an annual rate of 2.1% since 2005. However, the OMI SO_2_ over most regions showed an overall decrease before the rising peak occurred around 2007, despite a relatively large interannual variation. The average annual concentrations of OMI SO_2_ decreased by 60.6%, 59.2%, 48.7%, and 69.2% in the BTH, YRD, PRD, and SCB in 2017, respectively, compared to levels in 2005. Unlike primary pollutants such as NO_2_ and SO_2_, the HCHO column over central and eastern China showed an overall increasing trend of 13.7–27.0%. Note that these percentage changes are well within the 95% confidence interval (*P*-value less than 0.05), which were calculated based on the annual concentration relative to the year 2005.

### The marginal effect of individual meteorological variables on air pollutants

**Fig. 2:**
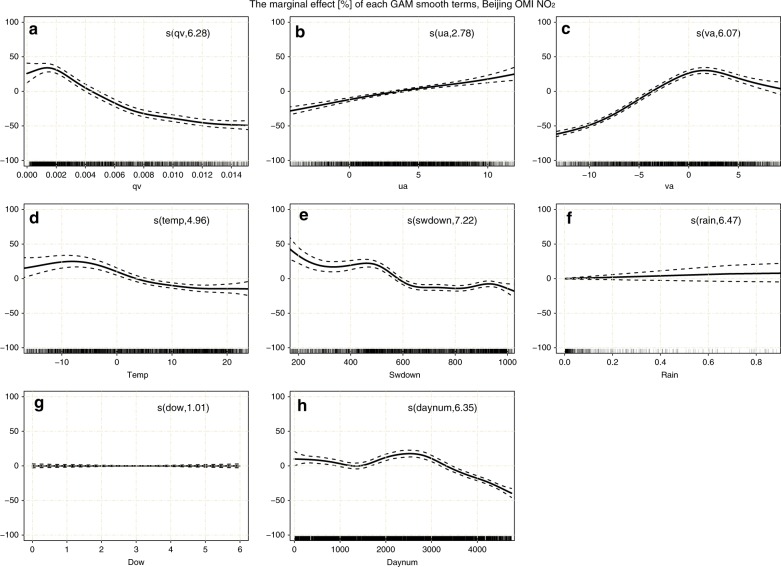
Plots of the marginal effects of individual covariates in the GAM on daily tropospheric NO_2_ in Beijing. **a–h** Covariates of *qv*, *ua*, *va*, *temp*, *swdown*, *rain*, *dow*, and *daynum* are shown in the panels. The EDF for the GAM smooth term is noted inside the bracket of the text. Each marginal effect is denoted by a solid line with a 95% confidence interval (dashed lines), and the vertical lines adjacent to the lower x-axis represent the distributions of these covariates

We selected four typical megacities, including Beijing, Shanghai, Guangzhou, and Chengdu (from BTH, YRD, PRD, and SCB, respectively), for the GAM analyses. The marginal effect of the smooth term S(*X*_*i*_) in the GAMs is calculated as $100\% \cdot [e^{S\left( {X_i} \right)} - 1]$, representing the relative contribution of the individual term to the overall response while other covariates are assumed to remain constant. Figure 2 and Figs. S2–12 illustrate the marginal effect of individual meteorological and temporal covariates, i.e., the water vapor mixing ratio (*qv*), zonal wind (*ua*), meridional wind (*va*), temperature (*temp*), downward shortwave solar radiation (*swdown*), precipitation (*rain*), day number (*daynum*), and day of the week (*dow*), for different trace gases and cities, respectively. Note that for each panel in the plots, the estimated degrees of freedom (EDFs) corresponding to the smooth term are noted inside the bracket of the text. An EDF of 1 indicates a linear effect. See the model details in the Materials and Methods section.

The reaction of water vapor with O (^1^D) atoms is a major source of tropospheric OH radicals, especially in the lower troposphere, where *qv* is large^2^. Therefore, water vapor may affect most reactive atmospheric pollutants through OH oxidation. An overall inverse relationship of tropospheric NO_2_ or SO_2_ with *qv* was found for most cities, which was possibly due to the reaction of the OH radical with NO_*x*_ or SO_2_. A positive relationship between HCHO and *qv* could possibly be related to secondary HCHO formation from the oxidation of VOCs^4^.

**Fig. 3:**
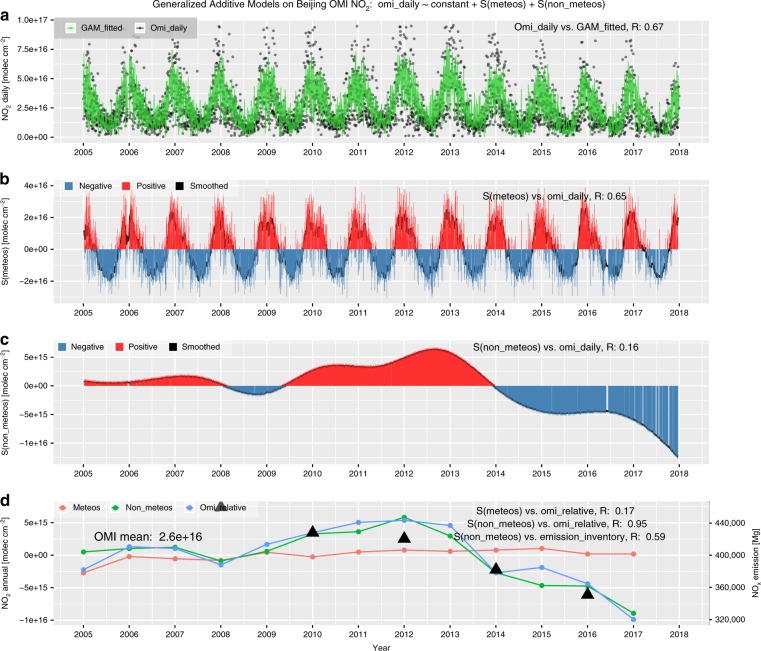
Time series components by GAMs of the tropospheric NO_2_ column over Beijing. **a** NO_2_ daily series of both OMI-measured and GAM-fitted data, as indicated by black dots and a green line, respectively. **b** The bar plot of the daily series of accumulated meteorological smooth terms, i.e., *S(meteos)*, where positive and negative *S(meteos)* are indicated with red and blue, respectively, while the black solid line denotes the smoothed series using a moving average with a window of 15 days. **c** same as **b** but for the non-meteorological terms, i.e., *S(non-meteos)*. **d** Interannual series of *S(meteos)*, *S(non-meteos)*, and relative NO_x_ variation compared to the overall mean, as shown with red, green, and blue dotted lines, respectively, while the triangular dots denote the MEIC NO_x_ emissions over Beijing, corresponding to the right y-axis

Local favorable wind conditions for air mass transport could have a determinant impact on air pollution levels. From the marginal effects in Beijing, we found that a southerly wind at a speed of 2 m s^–1^ could increase the tropospheric pollution level of NO_2_ by ~30%, that of SO_2_ by ~26%, and that of HCHO by ~4% compared to their overall means during 2005–2017 and that a northerly wind could effectively reduce air pollution levels by considerable amounts (see Figs. 3, S2–3). This finding is consistent with previous conclusions that a southerly wind aggravates haze pollution and that a northerly wind mitigates haze pollution^28,29^. Similar distinct positive correlations between a westerly wind and primary pollutants were also noticed in Shanghai (see Figs. S4–6). The wind effects indicated that the regional transport of pollutants plays an important role in the air quality of megacities. Compared to primary pollutants, the impact of wind speed on HCHO over these cities was much smaller. This could be explained by the short lifetime of tropospheric HCHO, which prevents the regional transport of its primary emissions^30^.

Furthermore, meteorological variables such as *temp*, *swdown*, and *rain* also play important roles in the formation, dispersion, and deposition of tropospheric pollutants^1^. The aggregated impacts of these variables could partially explain the seasonal variation in air pollutants, as seen by the marginal effect. Specifically, we found that there were almost no reductions in tropospheric NO_2_, SO_2_, and HCHO over these Chinese megacities during weekends, as seen by the marginal effect of *dow*. Such a weekly cycle was observed for developed countries such as the US and Japan^31,32^. The discrepancies may indicate the differences in the variation patterns of their major emissions.

## Discussion

In addition to the marginal effects of particular covariates, the time series accumulations of meteorological and non-meteorological (i.e., temporal) smooth terms are compared for these megacities. Figure 3 presents comparisons of the accumulated daily or annual series of meteorological and non-meteorological smooth terms during GAM modeling on OMI NO_2_ in Beijing, as indicated by *S(meteos)* and *S(non-meteos)*, respectively. Similar results were also shown for other gaseous pollutants over these four megacities in Figs. S13–21.

For OMI NO_2_ over these megacities, it was found that *S(meteos)* generally agreed well with the daily NO_2_ variations, with correlation coefficients (*R*) within 0.45–0.65. However, *S(non-meteos)* showed a lower frequency of variations and poor correlations with daily OMI NO_2_ (*R* = 0.16–0.29). Seasonally, *S(meteos)* varies between the maximum in winter and minimum in summer, which is consistent with the NO_2_ concentration. However, for interannual variability, *S(non-meteos)* generally coincides well with the measured OMI NO_2_ variations (*R* = 0.95–0.98), which is much better than *S(meteos)*, with an R smaller than 0.17 (see Figs. 3, S13–15). In addition, the magnitudes of the interannual variations in *S(non-meteos)* are 2.93–3.94 times larger than those in *S(meteos)* for these megacities. These statistical findings indicated that synoptic meteorological conditions dominate the short-term scale variability in tropospheric NO_2_, especially for megacities, with stronger seasonality in the mid-high latitudes, while the long-term or interannual NO_2_ variations are dominated by non-meteorological causes. Similar regular patterns were also found for SO_2_ and HCHO.

Given that the *S(non-meteos)* components have already been largely isolated from the meteorological influences, we further examined the ability of *S(non-meteos)* as an indicator of the anthropogenic causes of the ambient concentrations of air pollutants. For NO_2_ in Beijing, an overall high correlation was found between *S(non-meteos)* and NO_*x*_ emission inventory data from both bottom-up (*R* = 0.59, with the MEIC emission inventory^33^) and top-down (*R* = 0.72, with the OMI-derived emission inventory^34^; see Fig. S22) estimates. This suggests that *S(non-meteos)* could denote the variation in annual NO_x_ emissions by penalized regression splines for temporal covariates during GAM NO_2_ modeling. For SO_2_ and HCHO in these megacities, *S(non-meteos)* also generally correlated well with the emission inventories (see Figs. S13–21). This indicated that *S(non-meteos)* can be used to present the influence of anthropogenic emissions to some extent. Due to the complicated chemical process of different HCHO species in the atmosphere, the correlation coefficients between *S(non-meteos)* and VOC emission amounts varied over a large range.

Based on these GAM results, we can conclude that the downward trend in tropospheric NO_2_ in Beijing during 2012–2017 could be largely explained by the NO_x_ emission reductions due to the strict NO_x_ emission controls in the industrial sector and on vehicles since the APPCAP was issued in 2013^33,35^. Similar sharp decreases in NO_2_ *S(non-meteos)* were also found for Chengdu and Shanghai before the increase to its maximum in 2011 and 2012. However, a continuous reduction in NO_2_ *S(non-meteos)* occurred in Guangzhou in the PRD since 2007, indicating the effectiveness of stricter and earlier NO_*x*_ emission controls during the 11th Five-Year-Plan (2006–2010) in Guangdong Province^36^. Overall, local and nationwide efforts such as the APPCAP and other air quality policies have achieved a considerable reduction in anthropogenic NO_*x*_ emissions and therefore significantly improved air quality in these cities.

**Fig. 4:**
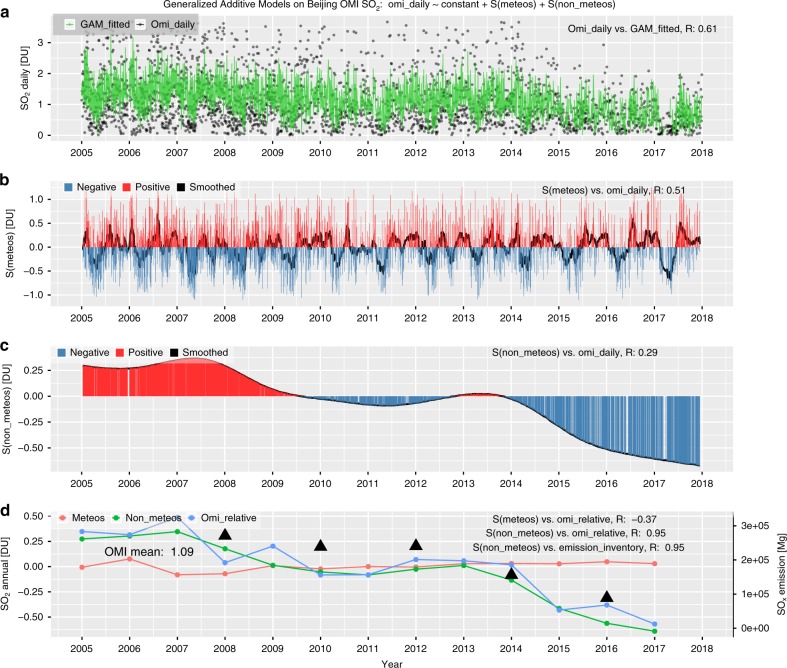
Similar to Fig. 3 but for tropospheric SO_2_ in Beijing. **a** SO daily series of both OMI-measured and GAM-fitted data, as indicated by black dots and a green line, respectively. **b** The bar plot of the daily series of accumulated meteorological smooth terms, i.e., S(meteos), where positive and negative S(meteos) are indicated with red and blue, respectively, while the black solid line denotes the smoothed series using a moving average with a window of 15 days. **c** Same as **b** but for the non-meteorological terms, i.e., S(non-meteos). **d** Interannual series of S(meteos), S(non-meteos), and relative SO_2_ variation compared to the overall mean, as shown with red, green, and blue dotted lines, respectively, while the triangular dots denote the MEIC SO_2_ emissions over Beijing, corresponding to the right *y*-axis

The sharp reductions in both OMI SO_2_ and *S(non-meteos)* over these cities were found during 2012–2017 (see Figs. 4, S16–18), which was possibly attributed to a combination of factors, such as the upgraded emission standards published during the 12th Five-Year Plan (2011–2015), deployment of flue gas de-sulfurization at coal-fired power plants, stricter emission controls during the APPCAP, and declines in coal consumption^37,38^. In addition, a smaller reduction during 2008–2010 was noticed for Beijing, Shanghai, and Guangzhou, which was possibly caused by the economic recession and local emission regulations for important events such as the Beijing 2008 Summer Olympics and the Expo 2010 in Shanghai, China.

**Fig. 5:**
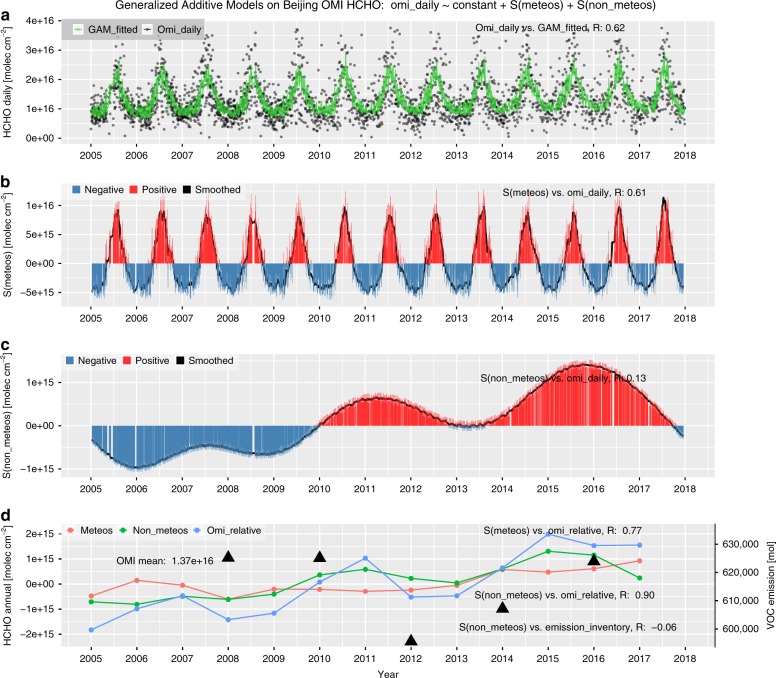
Similar to Fig. 3 but for tropospheric HCHO in Beijing. **a** HCHO daily series of both OMI-measured and GAM-fitted data, as indicated by black dots and a green line, respectively. **b** The bar plot of the daily series of accumulated meteorological smooth terms, i.e., S(meteos), where positive and negative S(meteos) are indicated with red and blue, respectively, while the black solid line denotes the smoothed series using a moving average with a window of 15 days. **c** Same as **b** but for the non-meteorological terms, i.e., S(non-meteos). **d** Interannual series of S(meteos), S(non-meteos), and relative HCHO variation compared to the overall mean, as shown with red, green, and blue dotted lines, respectively, while the triangular dots denote the MEIC VOCs emissions over Beijing, corresponding to the right *y*-axis

For HCHO, an overall increasing trend was found for these cities, especially during recent years since 2012 or 2013 (see Figs. 5, S19–21). In contrast to NO_2_ and SO_2_, which experienced sharp reductions recently, an unexpected HCHO increase was noted during 2013–2017 in Beijing. This could be explained by increases in interannual HCHO *S(non-meteos)* in the GAMs, which was also evidenced by the VOC emission inventory data^33^. This finding emphasises the vital role of VOC emission regulations when controlling HCHO pollution in these megacities.

**Fig. 6:**
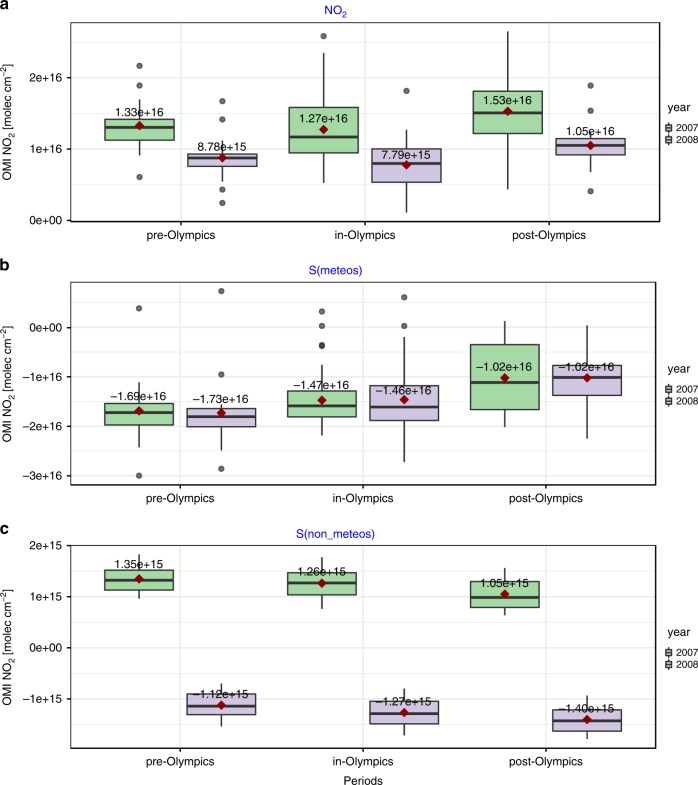
The box plots of period-averaged components of GAM NO_2_ modeling before, during, and after the Beijing Summer Olympics in 2008. The GAM components, such as OMI NO_2_, *S(meteos)*, and *S(non-meteos)*, are presented in **a**, **b**, and **c**, respectively. Within each box plot, the lower and upper bounds correspond to the 25th and 75th quartiles, while the solid line represents the median; the top and bottom whiskers extend from the hinges to the largest values by no more than 1.5* IQR (interquartile range) from the hinges. The mean values are noted by red square points with numbers. The black points outside the whisker are outliers

Apart from interpreting the long-term air quality trends, we also investigated the short-term impact of emissions change and synoptic meteorology on air quality changes. For example, we compared the measured concentrations, *S(non-meteos)* and *S(meteos)* of NO_2_ for the periods before, during, and after the Beijing 2008 Summer Olympics (see Fig. 6). The NO_2_ concentration significantly decreased compared with the same periods during the previous year, and such reductions could be largely attributed to the decrease in *S(non-meteos)*, i.e., emission reductions due to regulations in the industrial and vehicle sectors. Compared to the same period in 2007, NO_2_ VCDs and *S(non-meteos)* during the Beijing Olympics decreased by 4.9 × 10^15^ and 2.5 × 10^15^ molecules cm^–2^ (with *P*-values of the two sample *T*-tests less than 0.05), respectively, while *S(meteos)* decreased by 0.1 × 10^15^ molecules cm^–2^ (with a *P*-value of 0.2). Similar reductions in *S(non-meteos)* for other trace gases and those for the Guangzhou 2010 Asian Games are shown in Figs. S23–27. We can conclude that emission reductions play a dominant role during air pollution, controlling air quality during these important events, despite unfavorable meteorological conditions.

In summary, the recent declines in primary pollutants such as NO_2_ and SO_2_ could be attributed to reductions in NO_x_ and SO_2_ emissions due to the effective emission regulations and other air quality policies, especially after the APPCAP was implemented in 2013. In contrast to primary pollutants, the opposite trends in HCHO during recent years may encourage the need to control the anthropogenic emission sources of VOCs. Moreover, the variations in these important aerosol precursors significantly affected the temporal trends in fine particles (PM_2.5_). For example, a slight decrease in PM_2.5_ during 2006–2012 was indicated by satellite aerosol optical depth data and surface observations^39,40^ and was possibly caused by the onset of SO_2_ emissions control around 2007. The following sharp decrease in PM_2.5_ concentration during 2012–2017 could be possibly caused by the trend reversal in NO_2_ in 2011 and the effective emission reductions in other aerosol precursors, such as SO_2_ and NH_3_ (ammonia), due to the APPCAP^41^. This study provides novel insight into natural and human factors affecting air quality evolution over eastern China and will be further extended by satellite spectral measurements with higher spatial resolution from newly launched space-borne instruments, such as TROPOMI^42^ and EMI^43^.

## Materials and methods

### Satellite UV-Vis spectroscopy

The OMI is a nadir viewing push-broom spectrometer onboard NASA’s EOS Aura spacecraft in a low-earth polar orbit, measuring the entire solar spectrum from 270 to 540 nm at a moderate resolution of ~0.5 nm^12^. The OMI generally shows stable performance in radiometric and spectral calibrations since its launch in 2005, providing continuous spectroscopic measurements for Earth’s atmospheric components during its entire mission time^26^.

**Fig. 7:**
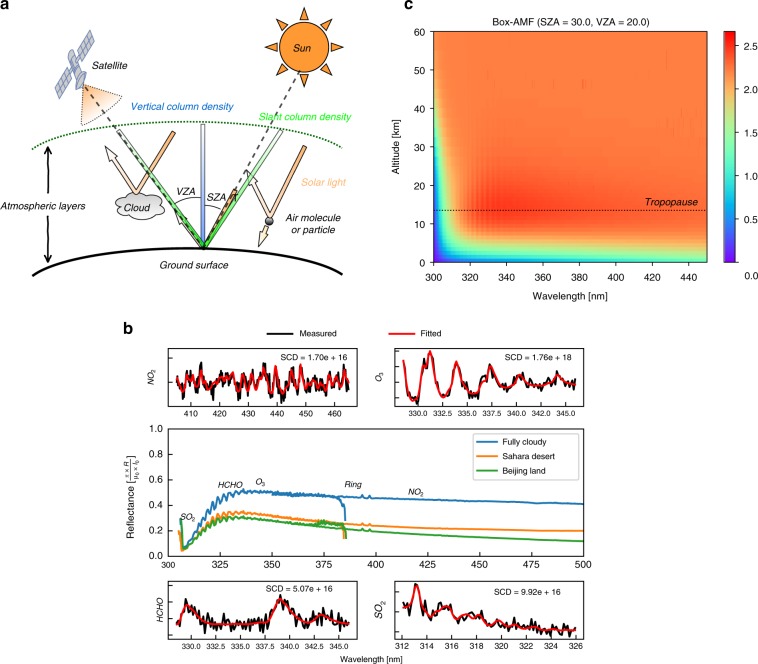
Illustration of the satellite spectroscopy principle of trace gas retrieval. **a** The viewing geometry of a typical satellite UV-Vis instrument and atmospheric radiation transfer processes, including absorption, reflection, and scattering. The definitions of the satellite solar zenith angle (SZA) and viewing zenith angle (VZA) and the slant column density (SCD) and vertical column density (VCD) of trace gases are noted. **b** An example of OMI-measured top-of-atmosphere reflectance in the UV2 and VIS1 channels is shown in the middle panel under different surface conditions, and the DOAS fitting of the SCDs of NO_2_, O_3_, HCHO and SO_2_ at different wavelength ranges are shown in four surrounding panels. **c** The altitude-resolved box AMF as a function of spectral wavelength. The tropopause height is denoted with a dotted line. The box AMF was calculated by the VLIDORT model for a satellite nadir viewing geometry of SZA = 30°; VZA = 20°; surface albedo of 0.075; and typical atmospheric profiles of pressure, temperature, O_3_ and NO_2_ from the U.S. Standard Atmosphere for mid-latitude summer

Figure 7a illustrates a typical observing geometry of a space-borne UV-Vis spectrometer that receives solar photons backscattered by air molecules or particles and reflected by surfaces and clouds. By numerically modeling the measured satellite spectra, information on the abundances of trace gases and particles and surface conditions can be effectively obtained. In the UV-Vis range, thermal emissions can be ignored, and the scattering of trace gases spectrally varies much slower than the absorption, as shown from the top-of-atmosphere reflectance spectra for different surface types in Fig. 7b. Therefore, the absorption of trace gases with high-frequency structures could be well distinguished in the observed spectra.

The atmospheric components can be retrieved from the satellite measurements in a simplified way by solving the Beer-Lambert’s law equation on radiative transfer. However, some inverse problems are usually ill-posed, which is mainly due to nonlinear effects from instrument calibration errors and the ring effect. Typically, several algorithms are developed to resolve these problems, including nonlinear least-square fitting, principal component analysis, optimal estimation (OE), and neural networks. Figure 7b gives an example of OMI spectral fitting of the slant column densities (SCDs) for NO_2_, HCHO, and SO_2_ using the state-of-the-art Differential Optical Absorption Spectroscopy (DOAS) technique^44^.

The absorption of the target trace gas in measured atmospheric radiation depends not only on the gas abundance but also on the average length of the path that a photon travels through in the atmosphere. Thus, numerical simulations by the atmospheric radiative transfer model (RTM) are needed to calculate the effective photon transfer path compared to a single vertical path, i.e., the so-called air mass factor (AMF), which converts the SCDs into vertical column densities (VCDs). The AMF is usually formulated by the integral of the vertical profile of the target gas weighted by altitude-dependent scattering weights. The uncertainty in AMF calculations is one of the dominant error sources for tropospheric trace gas retrievals^45^ (see the illustration of the SCD, VCD and altitude-dependent AMF in Fig. 7a, c). In addition, more realistic considerations in the radiative simulation of satellite-measured spectra, e.g., the ring effect, polarization and surface reflectance anisotropy, could effectively improve the accuracy and precision of trace gas retrievals, especially for weak absorbers such as SO_2_ and HCHO.

### Description of trace gas retrieval

The tropospheric retrieval of NO_2_ and HCHO followed a two-step approach, in which the spectral fitting of SCDs and the AMF calculations with the RTM were separated^44^. For SO_2_, an OE method was implemented by iteratively minimizing the differences between the measured and simulated spectra and between the retrieved and a priori state vectors using the RTM as the forward model^46,47^. The main algorithm improvements include the use of local-updated a priori information from the regional chemical transport model, direct RTM calculations instead of interpolations by a look-up table, and optimized configuration parameters such as instrument slit functions and gas cross-sections^48^.

The algorithm details for the NO_2_, SO_2_, and HCHO retrievals are provided in the Supplementary Information. Note that the data used in this study were screened first by cloud fraction, retrieval error and related quality flags for each satellite ground pixel (see Supplementary Information). Compared with the operational OMI trace gas products, our trace gas retrievals showed improved consistencies with independent ground-based measurements from MAX-DOAS and LiDAR over eastern China^49^.

### The GAMs

To further quantify the impact factors for air quality trends, a statistical fitting approach based on GAMs^27^ was implemented. GAMs make use of penalized smoothing splines, which address the complex non-linearity existing in air quality research. Meteorological variables were obtained from the NCEP FNL global meteorological dataset and then simulated at a horizontal resolution of ~20 km by using the WRF (Weather Research and Forecasting) model.

The GAM associated with daily series of pollutant concentrations can be written with the following equation:

$$\log \left( y \right)\sim \beta + \mathop {\sum }\limits_i^n S\left( {X_i} \right) + \varepsilon$$

where *y* is the daily pollutant concentration, *β* is the constant mean of the response, *S*(*X*_*i*_) is the smoothing function term of the *i*th component of *n* total covariates, and *ε* is the fitting residual. Here, the covariates *X*_*i*_ included meteorological variables such as zonal wind (*ua*), meridional wind (*va*), water vapor mixing ratio (*qv*), downward shortwave solar radiation at the surface (*swdown*), precipitation (*rain*), and temperature (*temp*), as well as other temporal variables such as the day number (*daynum*) and day of the week (*dow*), to account for the short-term temporal persistence and control for temporal autocorrelation in the residuals. Note that *ua*, *va*, *qv*, and *temp* are selected at a pressure level of 850 hPa (~1.5 km altitude), representing the lower troposphere, which is where most air pollutants are located.

## Supplementary information

Supplementary information for

## Data Availability

The OMI Level 1 data are available on NASA’s webpage at https://disc.gsfc.nasa.gov/datasets?source=Aura%20OMI&processingLevel=1B. Meteorological datasets are available from the NCEP FNL webpage at https://rda.ucar.edu/datasets/ds083.2/. The MEIC emission inventory data are available from http://www.meicmodel.org/. The OMI trace gas data used in this study can be requested from the corresponding author (chliu81@ustc.edu.cn).
