# Supplementary material for: Satellite UV-Vis spectroscopy: implications for air quality trends and their driving forces in China during 2005–2017
Source: Light Sci Appl. 2019 Nov 13;8:100. doi: 10.1038/s41377-019-0210-6 (PMC6853934; doi:10.1038/s41377-019-0210-6)
Supplement: Supplementary file 1 — Supplementary information for [file 41377_2019_210_MOESM1_ESM.pdf]

Supplementary information for

Satellite UV-Vis spectroscopy: implications for air quality trends and their driving forces in China during 2005-2017

Chengxin Zhang[1,#], Cheng Liu[2,3,4,5,#,*], Qihou Hu[3,*], Zhaonan Cai[6,*], Wenjing Su[1], Congzi Xia[1], Yizhi Zhu[3], Siwen Wang[7], Jianguo Liu[3,4]

[1]School of Earth and Space Sciences, University of Science and Technology of China, Hefei, 230026, China
[2]Department of Precision Machinery and Precision Instrumentation, University of Science and Technology of China, Hefei, 230026, China
[3]Key Laboratory of Environmental Optics and Technology, Anhui Institute of Optics and Fine Mechanics, Chinese Academy of Sciences, Hefei, 230031, China
[4]Center for Excellence in Regional Atmospheric Environment, Institute of Urban Environment, Chinese Academy of Sciences, Xiamen, 361021, China
[5]Key Laboratory of Precision Scientific Instrumentation of Anhui Higher Education Institutes, University of Science and Technology of China, Hefei, 230026, China
[6]Key Laboratory of Middle Atmosphere and Global Environment Observation, Institute of Atmospheric Physics, Chinese Academy of Sciences, Beijing, 100029, China
[7]Multiphase Chemistry Department, Max Planck Institute for Chemistry, Mainz, 55128, Germany

[#]These authors contributed equally to this work

[*]Corresponding authors: Cheng Liu (chliu81@ustc.edu.cn), Qihou Hu (qhhu@aiofm.ac.cn), and Zhaonan Cai (caizhaonan@mail.iap.ac.cn)

Supplementary Texts

On the OMI instrument

As seen from measured earthshine radiance as well as solar irradiance, the OMI instrument shows low optical degradation and high spectral stability over the mission time. OMI irradiances have degraded by 3-8% while radiances have changed by 1-2%[1]. The wavelength shift of the OMI instrument remains to be within 0.02 nm[1]. The Signal-to-noise ratio of OMI has been gradually decreasing over the years due to the expected CCD degradation[2]. The phenomenon called "row anomaly (RA)" occurred since 2007 and changed over time[1], which affects the level 1b data at all wavelengths for particular viewing directions or cross-track positions.
To overcome these addressed issues such as optical degradation and row anomaly, we have implemented specified methods during the retrieval of each trace gas. For example, a systematic cross-track biases were existed in the NO_2 SCDs retrieval, possibly caused by the

imperfect calibration in the solar irradiance. Therefore, a de-striping procedure was implemented to correct such stripe-like patterns[3]. And the optical degradation in solar irradiance and signal-to-noise ratio (SNR) significantly could affect the retrieval of weak absorbers such as HCHO and SO_2. For HCHO SCD fittings, nadir reference spectrum from radiance data was used instead due to the large uncertainty in the solar irradiance. For SO_2 retrieval, we applied "soft calibration" to OMI radiance, and thus the SO_2 fitting residual was largely reduced[4]. Note that the retrieval uncertainties of these gases are reported to be increasing slightly over the mission time, as described in Zara et al., 2019[5], due to the optical degradation. For the RA effect, we excluded all satellite RA-affected pixels during the re-gridding of level 2 data.

OMI NO_2 retrieval

During NO_2 SCDs retrieval, we generally follow the common DOAS configurations as suggested in QA4ECV NO_2 project[5]. The NO_2 spectral fit was selected in the wavelength range of 405-465nm and performed with the QDOAS software package[6]. For NO_2 AMF simulations, stratospheric and tropospheric NO_2 AMF were calculated pixel-by-pixel by the VLIDORT model at version 2.7[7]. During the RTM calculations, *a priori* NO_2 profile was obtained from the monthly GEOS-Chem simulations[8] at a resolution of 2 × 2.5°; and additional information such as cloud fraction, cloud top pressure, and surface albedo, was taken from the operational OMI O_2-O_2 cloud dataset[9]. During stratosphere-troposphere separation on the total NO_2 column, we estimated the stratospheric contribution from the total NO_2 column based on a modified reference sector method, i.e., the STREAM algorithm[10].

Finally, the tropospheric NO_2 VCDs per orbit were re-gridded to the Level 3 product with a resolution of 0.1× 0.1° over eastern China by using a novel P-Spline method[11]. Note that satellite pixels were filtered out first if satisfying any of the following rejection criteria: cloud radiance fraction larger than 0.3; root mean square (RMS) of the spectral fitting larger than 0.002; pixels affected by the row anomaly; solar zenith angle larger than 70°; and other quality flags.

OMI HCHO retrieval

For HCHO SCDs retrieval, we generally followed the nonlinear least-square fitting methods as described in González Abad et al., 2016[12], based on the so-called BOAS (basic optical absorption spectroscopy) approach. During the AMF calculations, monthly *a priori* profile of HCHO from the WRF-Chem model was used over China. See retrieval algorithm details in our previous study[13].

A constant value method[11] was also implemented during the HCHO re-gridding, with data screening criteria applied (similar to that of NO_2).

OMI SO_2 retrieval

For SO_2 retrieval, we implemented an OEM approach with full radiative simulations to directly retrieve SO_2 VCDs simultaneously with ozone profile[4,14,15]. Compared to the previous algorithm for the GOME-2 instrument[15], the following updates are included for OMI SO_2 retrieval: 1) fitting wavelength range is optimized at 312-326 nm; 2) monthly *a priori* atmospheric profile generated by the GEOS-Chem model was used[8].

A constant value method[11] was also implemented during the SO_2 re-gridding, with data screening criteria applied (similar to that of NO_2).

Ground-based validations

Our improved retrieval of trace gases including NO_2, HCHO and SO_2 have been widely used in ground-based MAX-DOAS measurements in eastern China, and shown overall better agreements compared to the operational OMI products[16-19]. Please refer to these literatures for detailed validation performances on the presented satellite data.

Supplementary Figures

Figure S1 showed the spatial distribution of linear regression slopes for annual NO_2, SO_2, and HCHO.

Figure S2-12 are similar to Fig. 3 in the manuscript, but are of the marginal effect plots for NO_2, SO_2, HCHO, over Beijing, Shanghai, Guangzhou, and Chengdu, respectively.

Figure S13-21 are similar to Fig. 4 in the manuscript, but are of the GAMs time series plots for NO_2, SO_2, HCHO over Shanghai, Guangzhou, and Chengdu, respectively.

Figure S22 are similar to Fig. 4d in the manuscript, but are for additional comparisons with top-down satellite NO_x and SO_2 emission inventory data[20,21] (estimated from the operational OMI NO_2 and SO_2 products, data available at http://www.globemission.eu/region_asia/datapage.php and http://www.globemission.eu/region_asia/ datapage_so2.php respectively, last access: 31 May 2019).

Figure S23-27 are similar to Fig. 7 in the manuscript, but are for the remaining box-plot comparisons for the Beijing 2008 Summer Olympics and Guangzhou 2012 Asian Games, respectively.

Reference

1 Schenkeveld, V. M. E. *et al.* In-flight performance of the Ozone Monitoring Instrument. *Atmos Meas Tech* **10**, 1957-1986, doi:10.5194/amt-2016-420 (2017).

2 Levelt, P. F. *et al.* The Ozone Monitoring Instrument: overview of 14 years in space. *Atmos Chem Phys* **18**, 5699-5745 (2018).

3 Boersma, K. F. *et al.* An improved tropospheric NO2 column retrieval algorithm for the Ozone Monitoring Instrument. *Atmos Meas Tech* **4**, 1905-1928, doi:10.5194/amt-4-1905-2011 (2011).

4 Cai, Z. N. *et al.* Characterization and correction of Global Ozone Monitoring Experiment 2 ultraviolet measurements and application to ozone profile retrievals. *J Geophys Res-Atmos* **117**, doi:10.1029/2011jd017096 (2012).

5 Zara, M. *et al.* Improved slant column density retrieval of nitrogen dioxide and formaldehyde for OMI and GOME-2A from QA4ECV: intercomparison, uncertainty

characterisation, and trends. *Atmos Meas Tech* **11**, 4033-4058, doi:10.5194/amt-11-4033-2018 (2018).
6 Fayt, C. *et al.* QDOAS Software user manual. *Belgian Institute for Space Aeronomy: Brussels, Belgium* **1** (2011).
7 Spurr, R. J. D. VLIDORT: A linearized pseudo-spherical vector discrete ordinate radiative transfer code for forward model and retrieval studies in multilayer multiple scattering media. *J Quant Spectrosc Ra* **102**, 316-342, doi:10.1016/j.jqsrt.2006.05.005 (2006).
8 Wang, S. W. *et al.* Growth in NOx emissions from power plants in China: bottom-up estimates and satellite observations. *Atmos Chem Phys* **12**, 4429-4447, doi:10.5194/acp-12-4429-2012 (2012).
9 Veefkind, J. P., de Haan, J. F., Sneep, M. & Levelt, P. F. Improvements to the OMI O2–O2 operational cloud algorithm and comparisons with ground-based radar–lidar observations. *Atmos Meas Tech* **9**, 6035-6049, doi:10.5194/amt-9-6035-2016 (2016).
10 Beirle, S. *et al.* The STRatospheric Estimation Algorithm from Mainz (STREAM): estimating stratospheric NO2 from nadir-viewing satellites by weighted convolution. *Atmos Meas Tech* **9**, 2753-2779, doi:10.5194/amt-9-2753-2016 (2016).
11 Kuhlmann, G., Hartl, A., Cheung, H. M., Lam, Y. F. & Wenig, M. O. A novel gridding algorithm to create regional trace gas maps from satellite observations. *Atmos Meas Tech* **7**, 451-467, doi:10.5194/amt-7-451-2014 (2014).
12 Abad, G. G. *et al.* Updated Smithsonian Astrophysical Observatory Ozone Monitoring Instrument (SAO OMI) formaldehyde retrieval. *Atmos Meas Tech* **8**, 19-32, doi:10.5194/amt-8-19-2015 (2015).
13 Su, W. *et al.* Characterization of ozone in the lower troposphere during the 2016 G20 conference in Hangzhou. *Sci Rep* **7**, 17368, doi:10.1038/s41598-017-17646-x (2017).
14 Liu, X., Bhartia, P. K., Chance, K., Spurr, R. J. D. & Kurosu, T. P. Ozone profile retrievals from the Ozone Monitoring Instrument. *Atmos Chem Phys* **10**, 2521-2537, doi:DOI 10.5194/acp-10-2521-2010 (2010).
15 Nowlan, C. R. *et al.* Retrievals of sulfur dioxide from the Global Ozone Monitoring Experiment 2 (GOME-2) using an optimal estimation approach: Algorithm and initial validation. *J Geophys Res-Atmos* **116**, doi:10.1029/2011jd015808 (2011).
16 Tan, W. *et al.* Tropospheric NO_2, SO_2, and HCHO over the East China Sea, using ship-based MAX-DOAS observations and comparison with OMI and OMPS satellite data. *Atmos Chem Phys* **18**, 15387-15402, doi:10.5194/acp-18-15387-2018 (2018).
17 Xing, C. Z. *et al.* Observations of the vertical distributions of summertime atmospheric pollutants and the corresponding ozone production in Shanghai, China. *Atmos Chem Phys* **17**, 14275-14289, doi:10.5194/acp-17-14275-2017 (2017).
18 Tan, W. *et al.* Estimation of winter time NOx emissions in Hefei, a typical inland city of China, using mobile MAX-DOAS observations. *Atmos Environ* **200**, 228-242 (2019).
19 Hong, Q. Q. *et al.* Ship-based MAX-DOAS measurements of tropospheric NO2, SO2, and HCHO distribution along the Yangtze River. *Atmos Chem Phys* **18**, 5931-5951, doi:10.5194/acp-18-5931-2018 (2018).
20 Ding, J. *et al.* Maritime NOx Emissions Over Chinese Seas Derived From Satellite Observations. *Geophys Res Lett* **45**, 2031-2037, doi:10.1002/2017gl076788 (2018).
21 van der A, R. J. *et al.* Cleaning up the air: effectiveness of air quality policy for SO2 and NOx emissions in China. *Atmos Chem Phys* **17**, 1775-1789, doi:10.5194/acp-17-1775-2017 (2017).

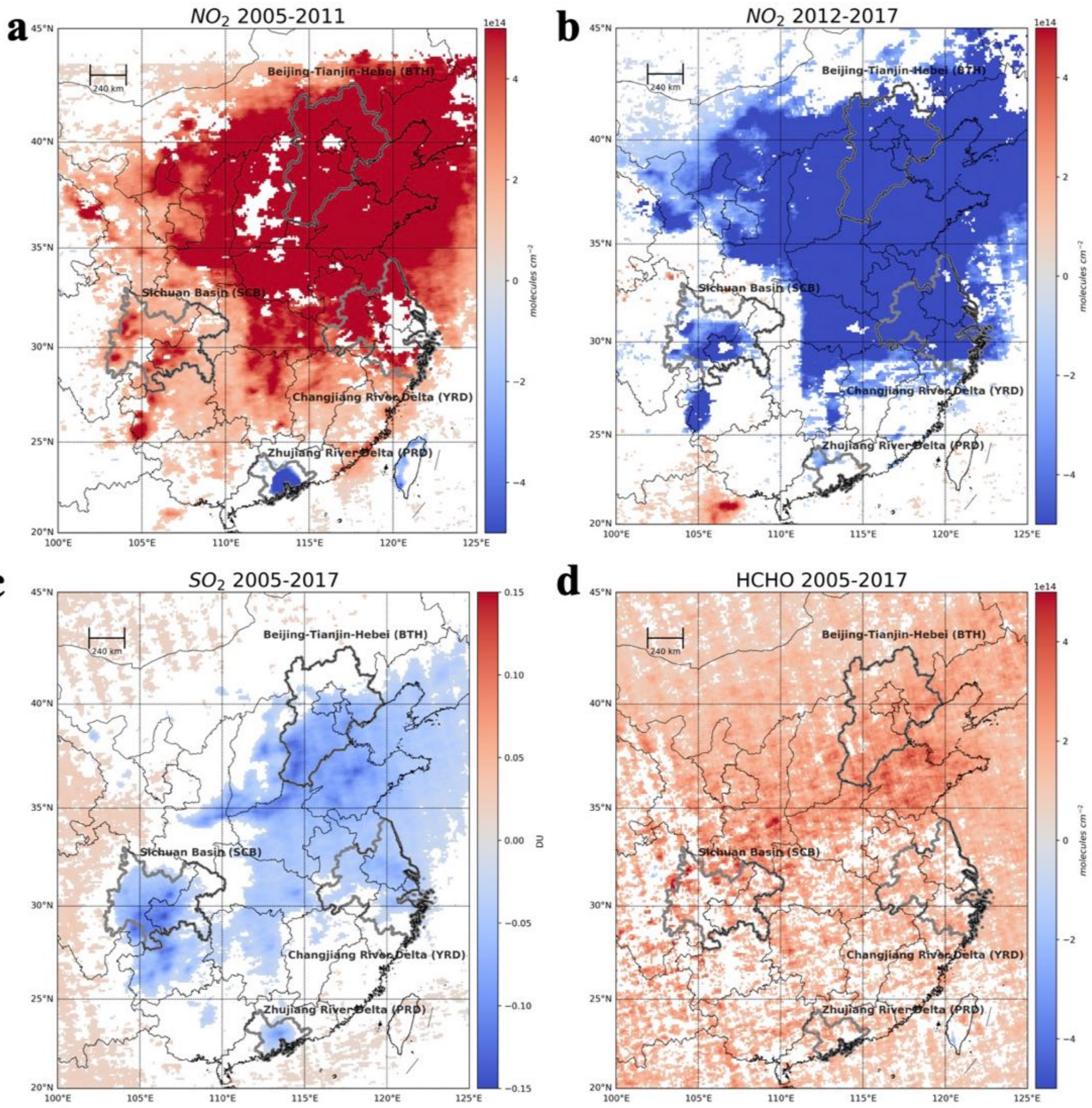

Figure S1. The spatial distribution of the annual rates (i.e., the linear-regression slopes) for annual NO_2, SO_2, and HCHO in each satellite ground pixel (at a resolution of 0.1 × 0.1°). The time periods of 2005-2011 and 2005-2017 for NO_2 were shown in **a**, **b**, respectively. And the whole periods of 2005-2017 for SO_2 and HCHO were shown in **c** and **d**, respectively. The slopes with P-value larger than 0.05 is filtered (shown as white).

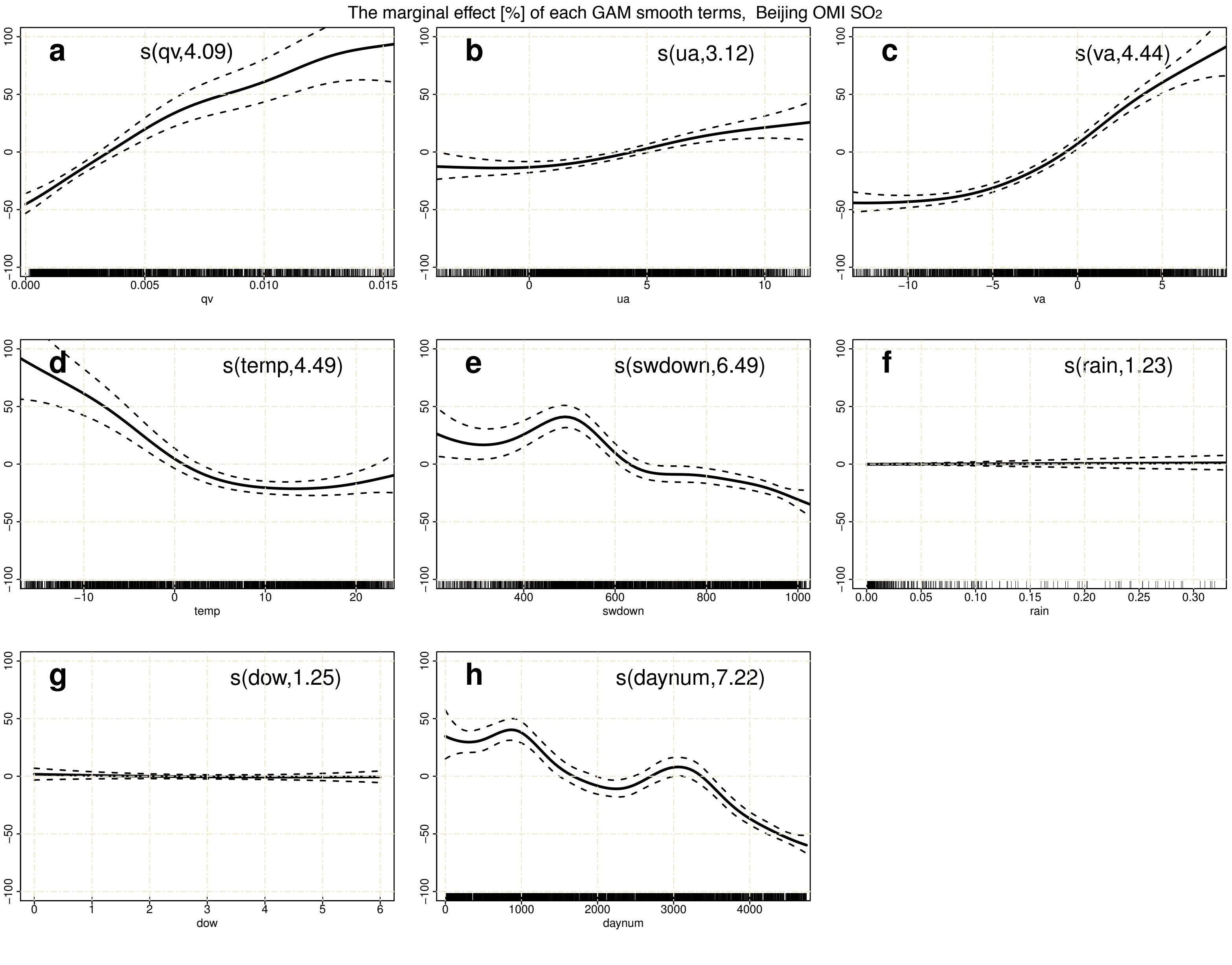

Figure S2. Similar to Fig. 2, but for SO_2 in Beijing

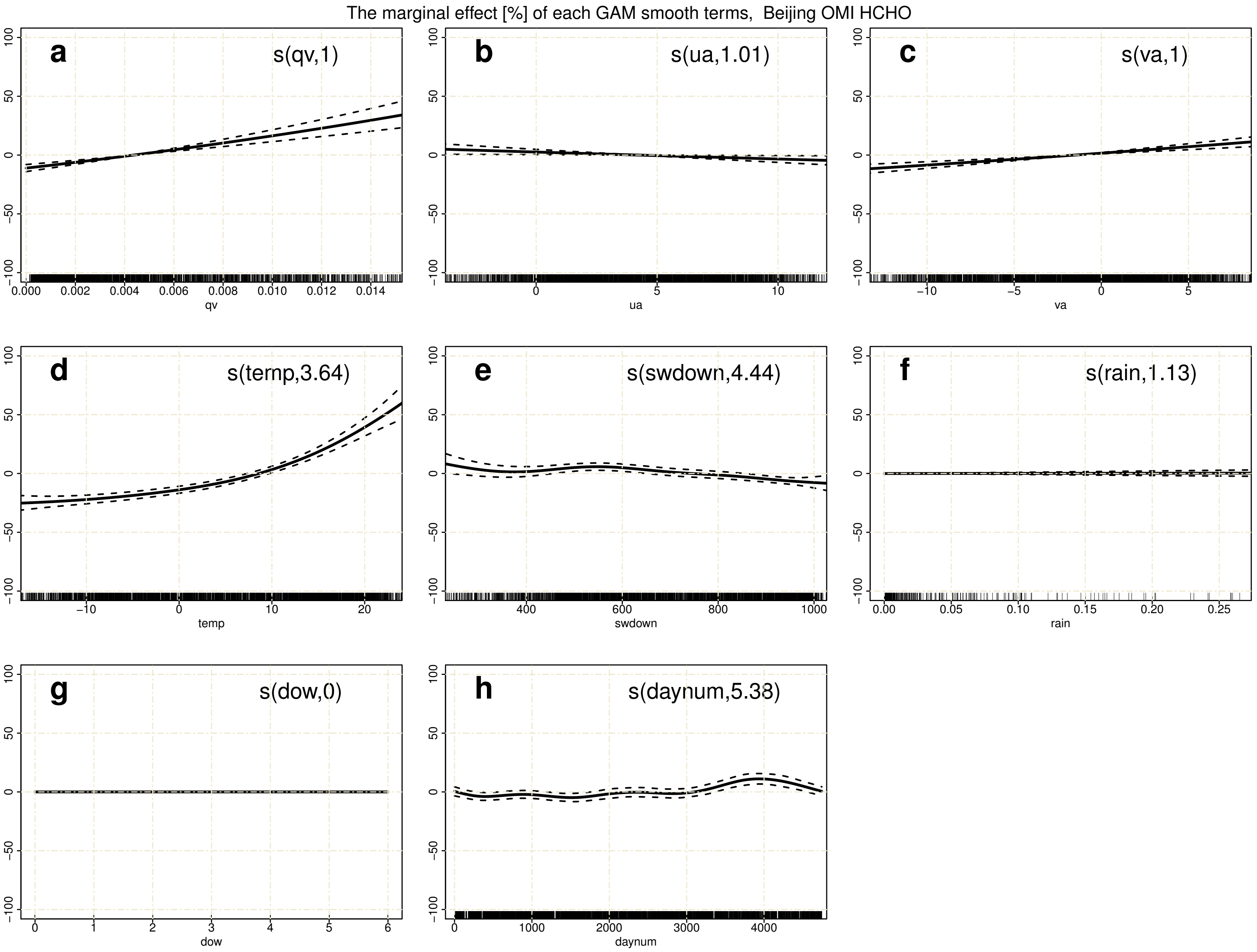

Figure S3. Similar to Fig. 2, but for HCHO in Beijing

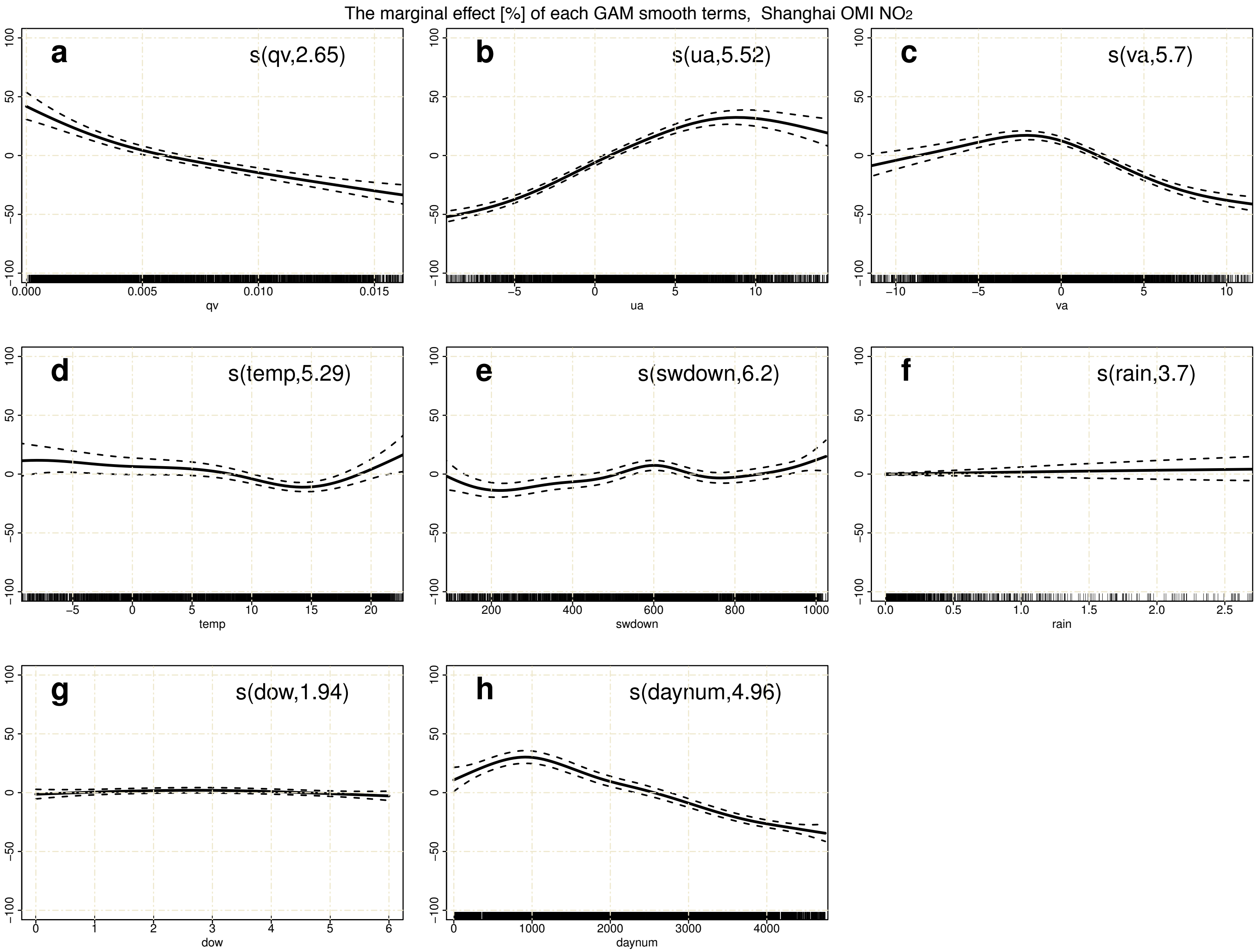

Figure S4. Similar to Fig. 2, but for NO_2 in Shanghai

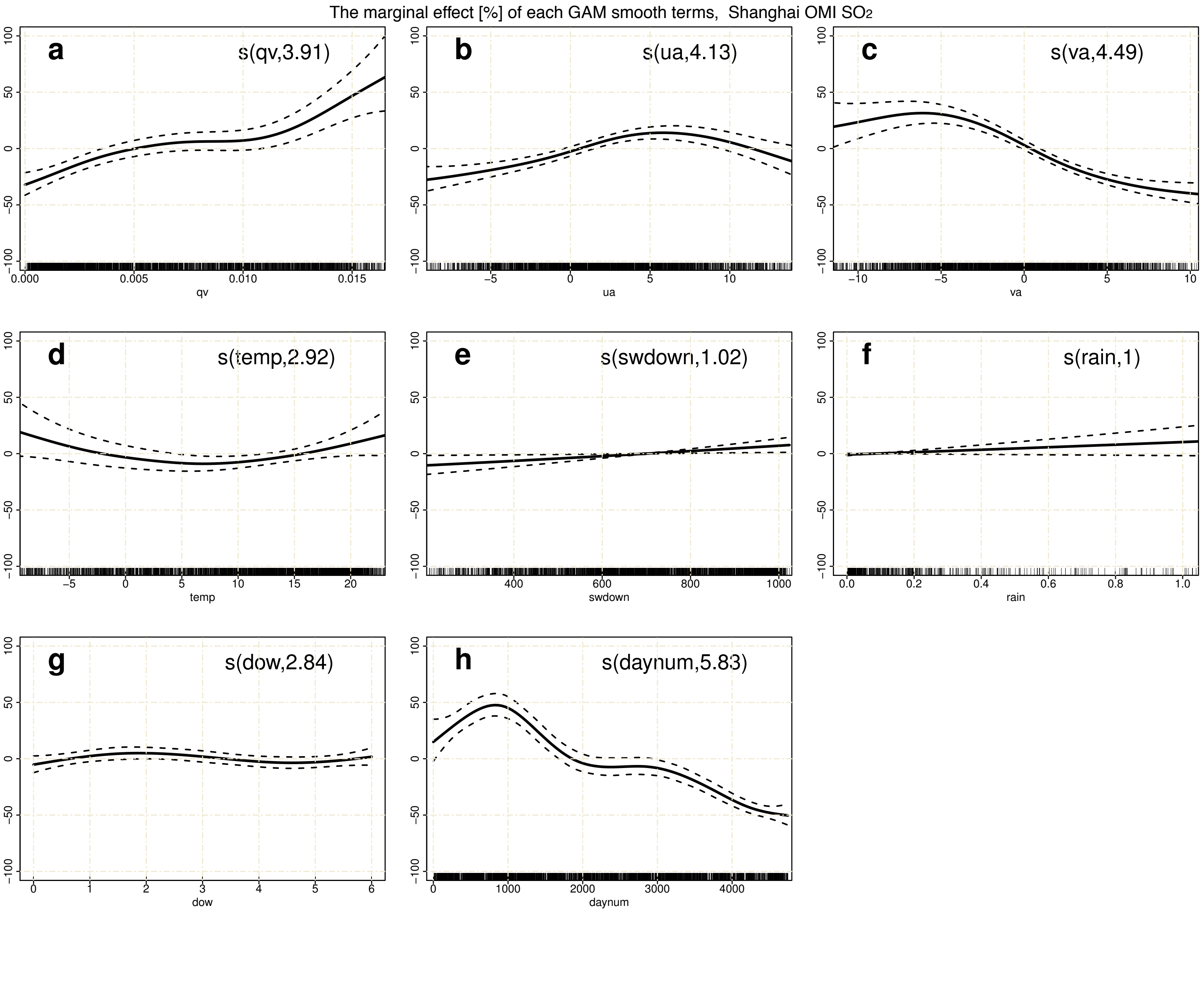

Figure S5. Similar to Fig. 2, but for SO_2 in Shanghai

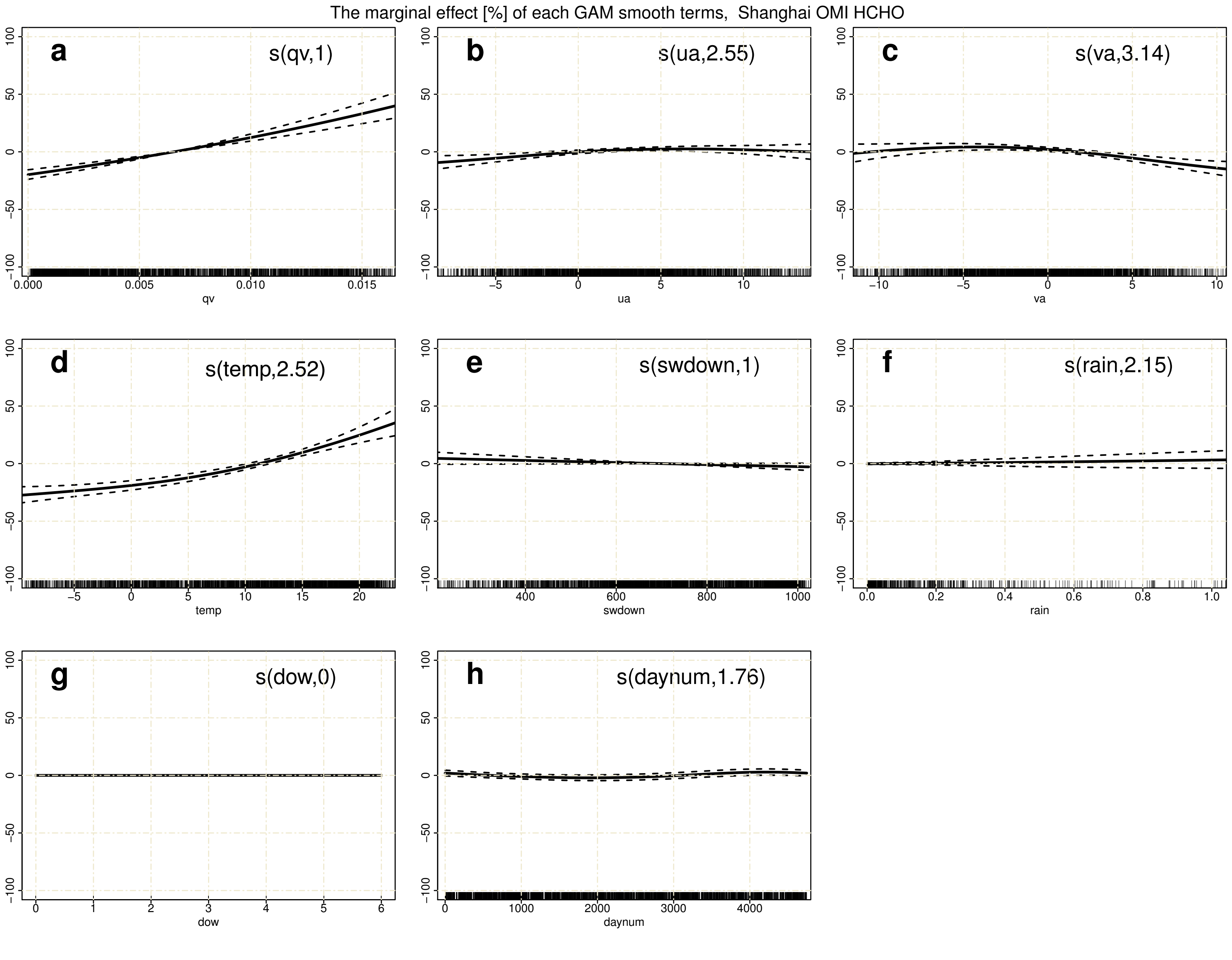

Figure S6. Similar to Fig. 2, but for HCHO in Shanghai

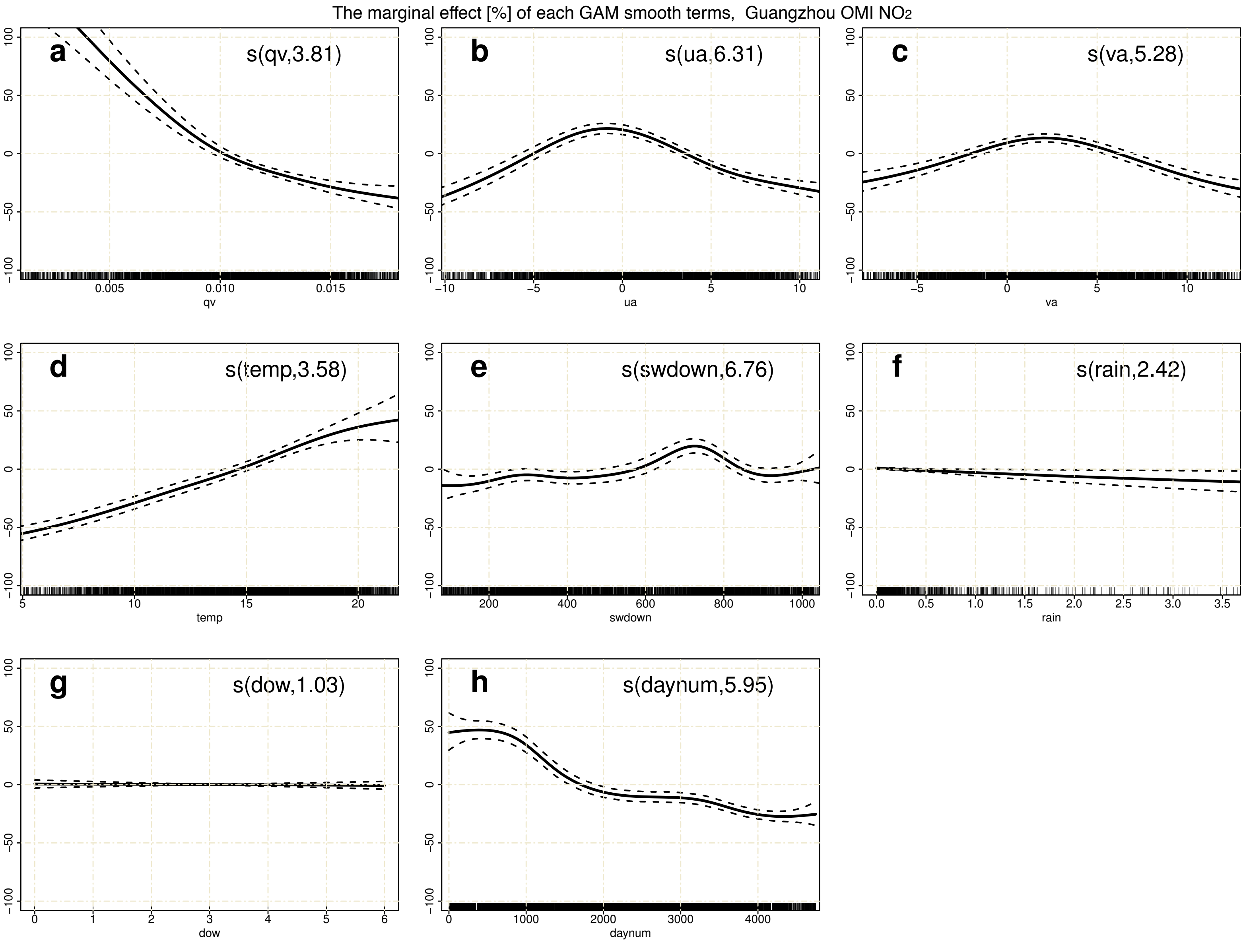

Figure S7. Similar to Fig. 2, but for NO_2 in Guangzhou

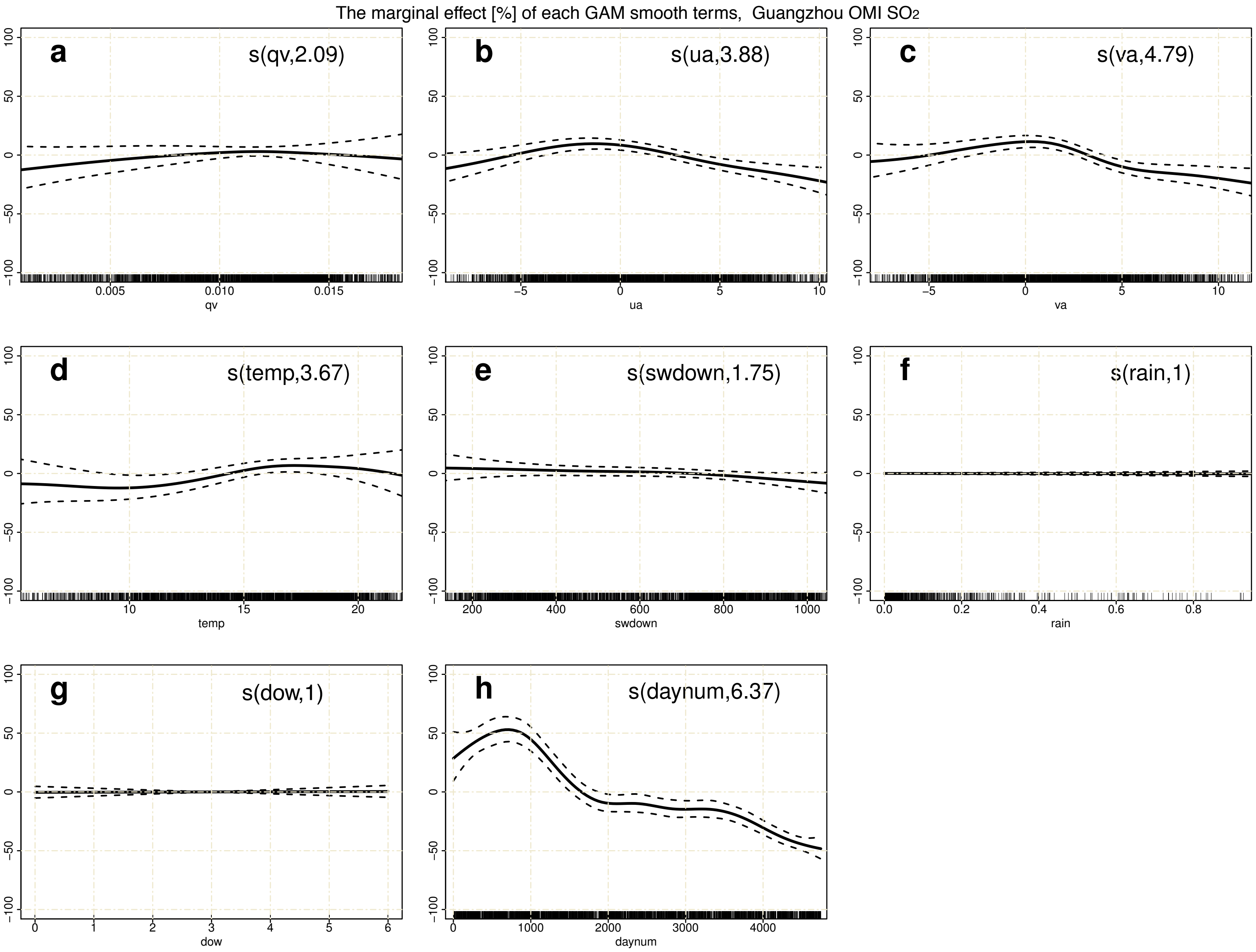

Figure S8. Similar to Fig. 2, but for SO_2 in Guangzhou

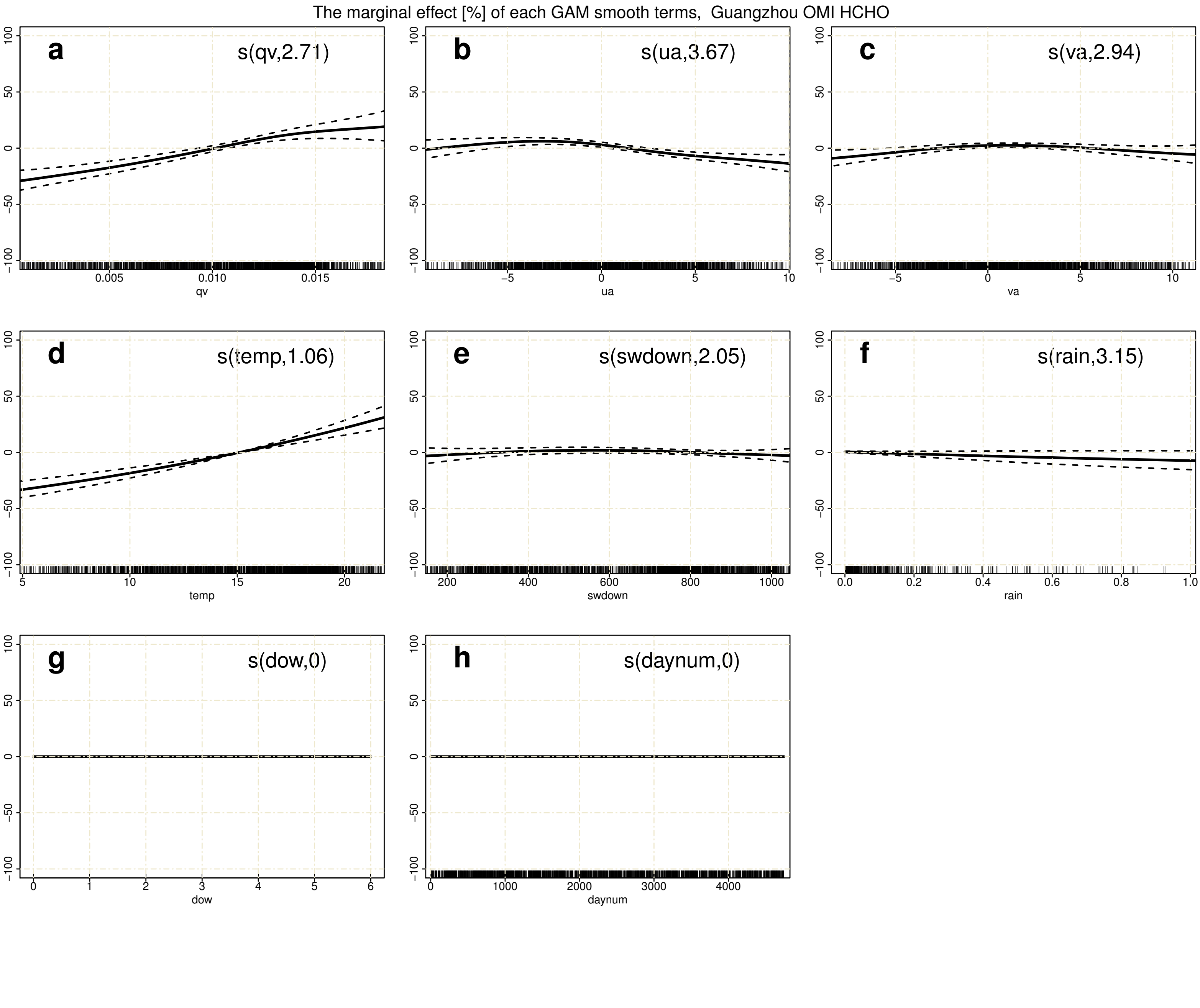

Figure S9. Similar to Fig. 2, but for HCHO in Guangzhou

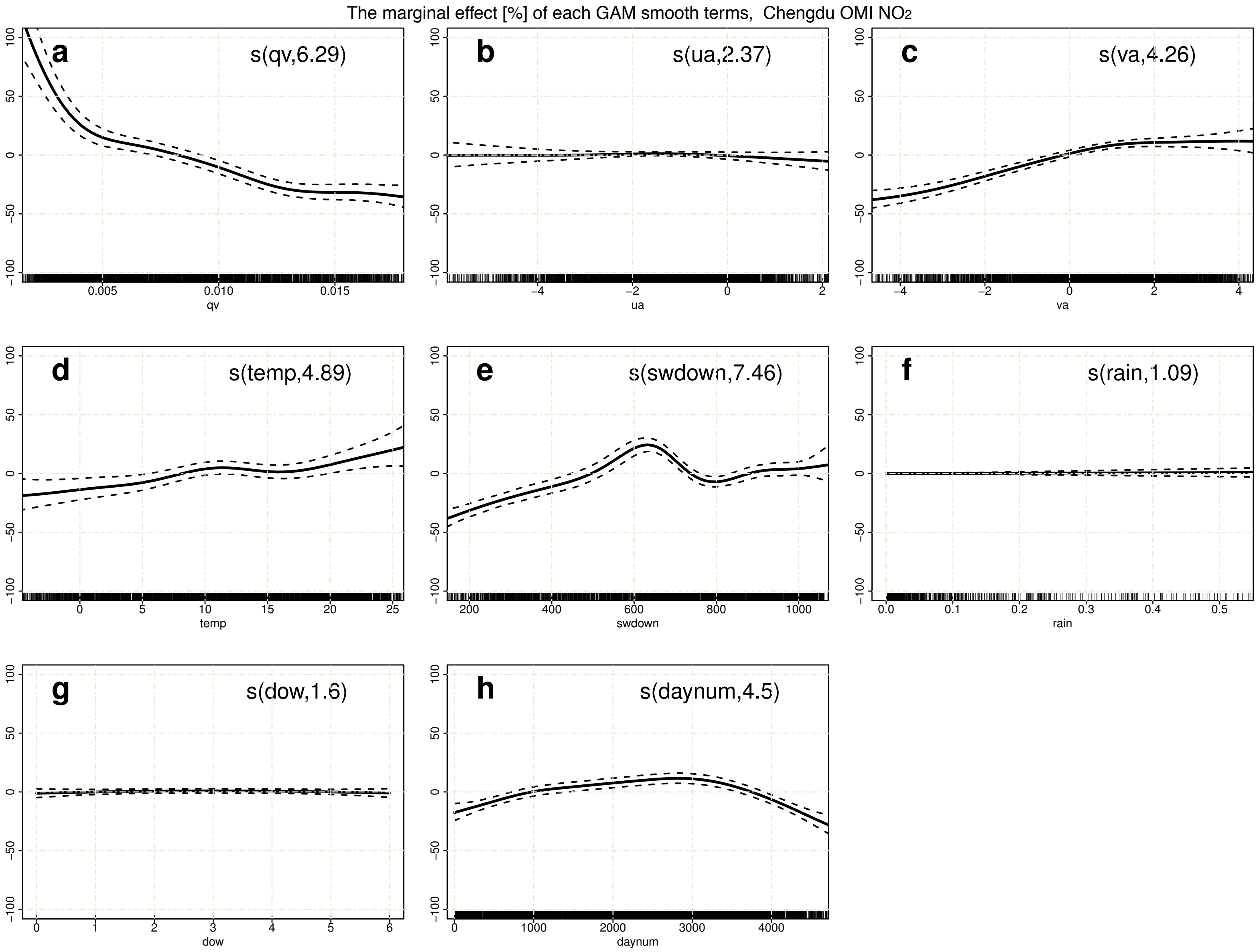

Figure S10. Similar to Fig. 2, but for NO_2 in Chengdu

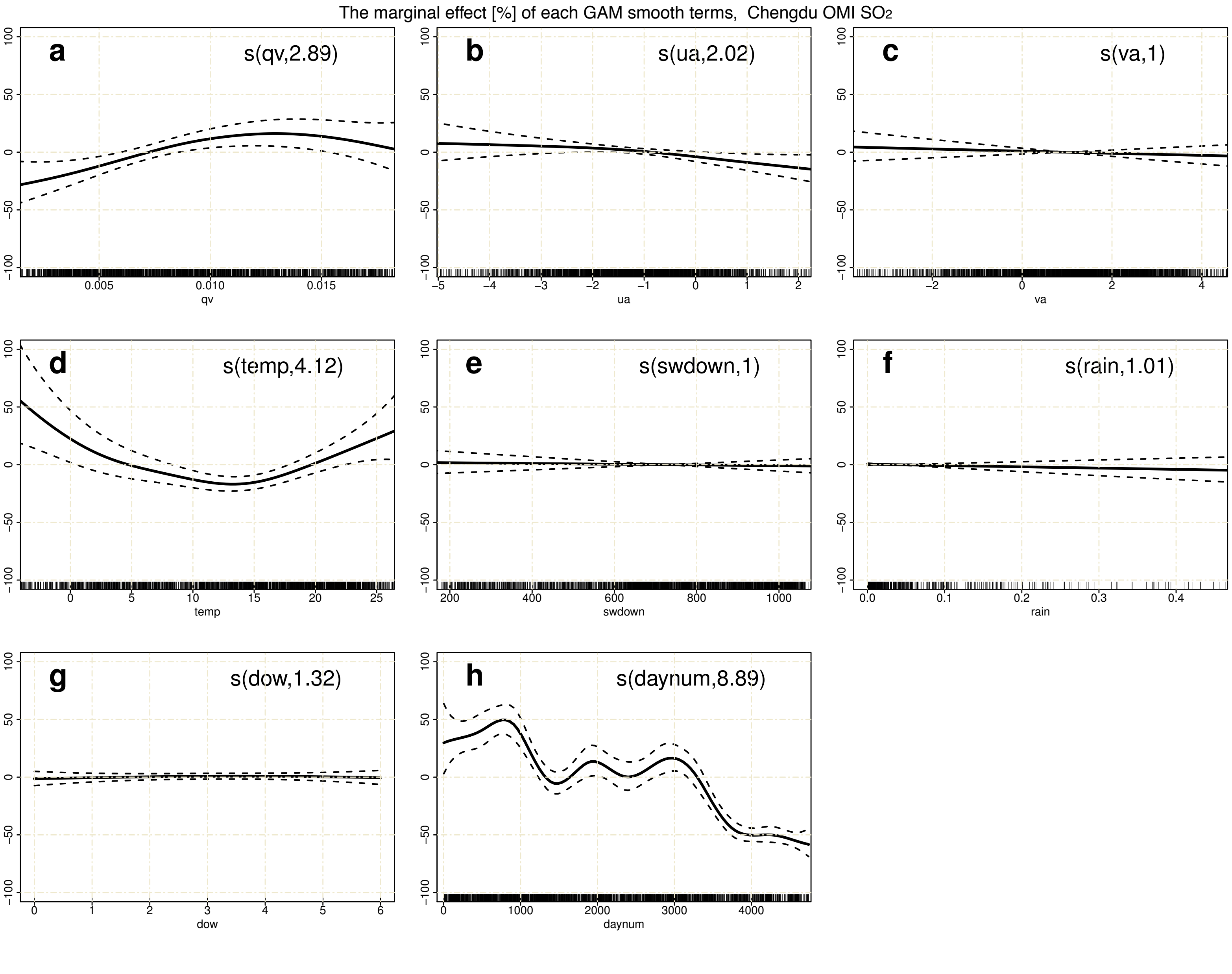

Figure S11. Similar to Fig. 2, but for SO_2 in Chengdu

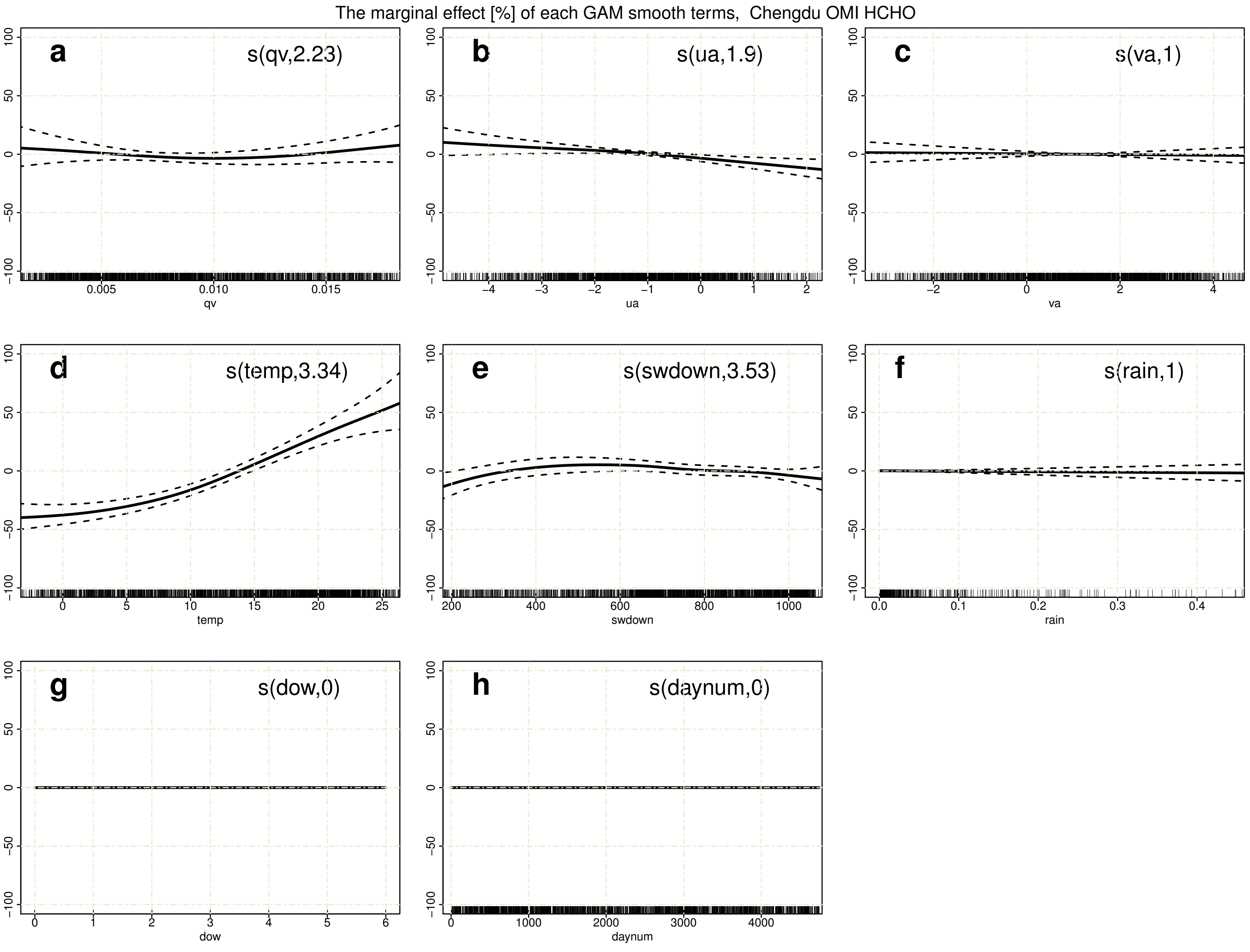

Figure S12. Similar to Fig. 2, but for HCHO in Chengdu

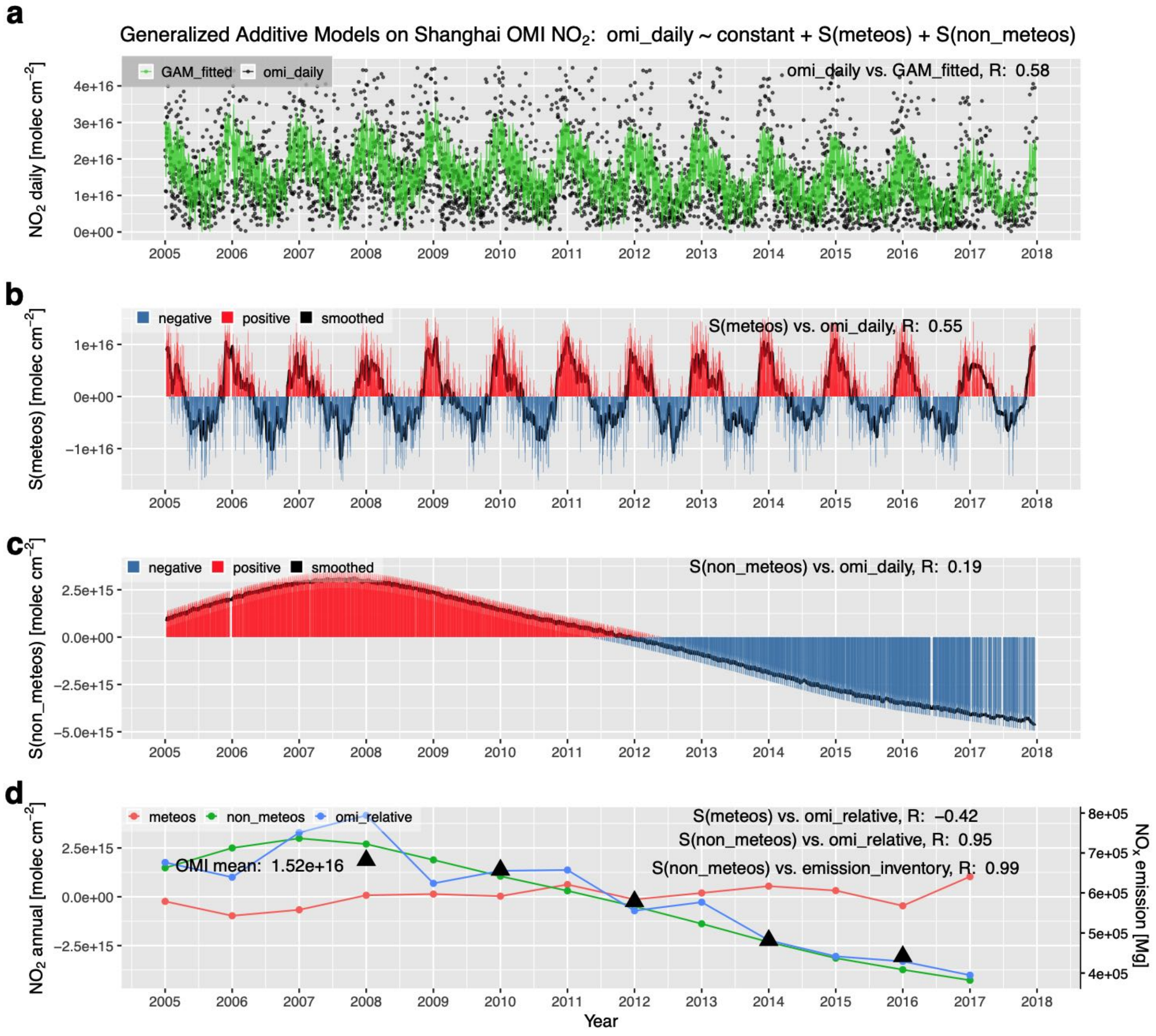

Figure S13. Similar to Fig. 3, but for NO_2 in Shanghai

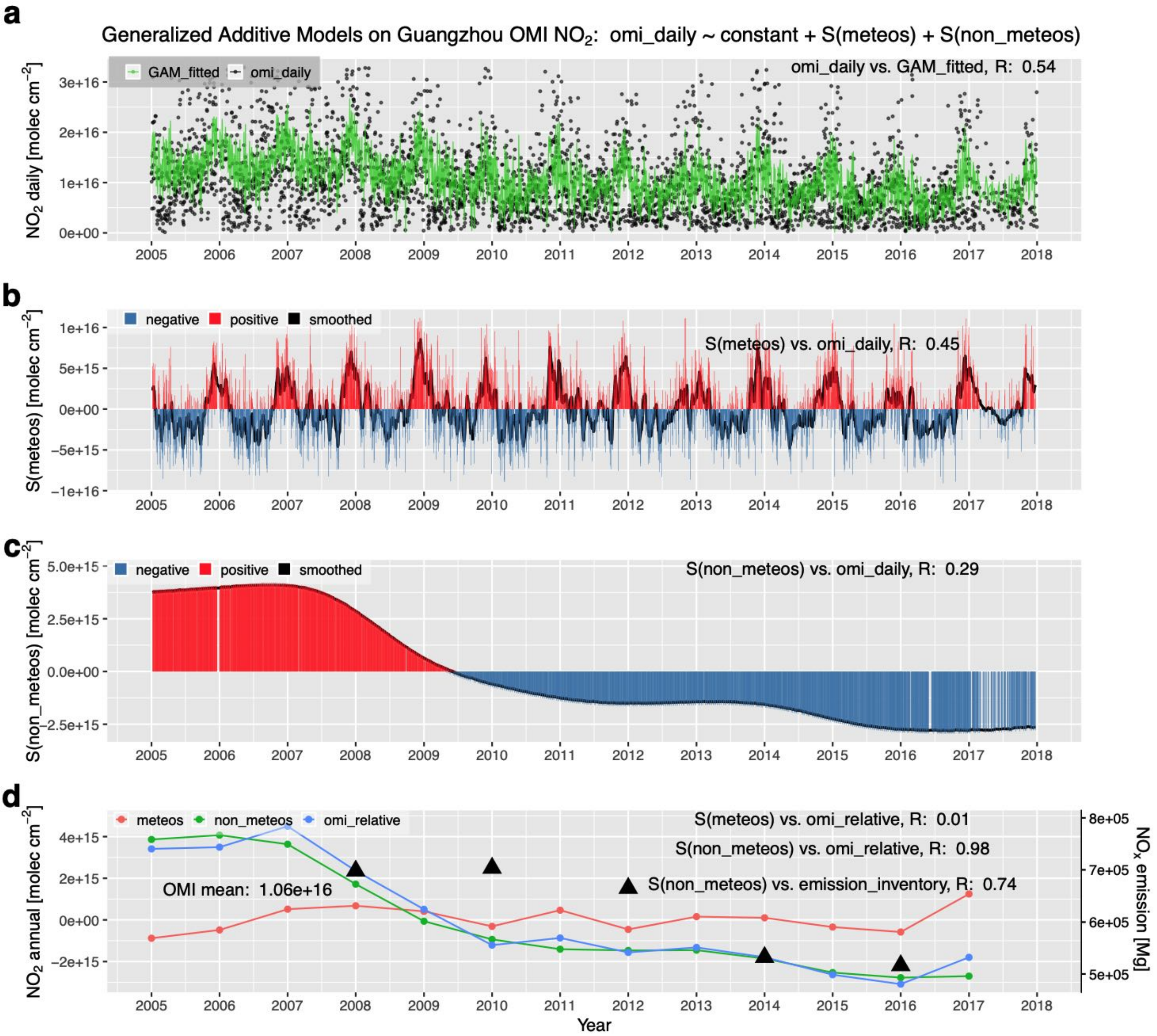

Figure S14. Similar to Fig. 3, but for NO2 in Guangzhou

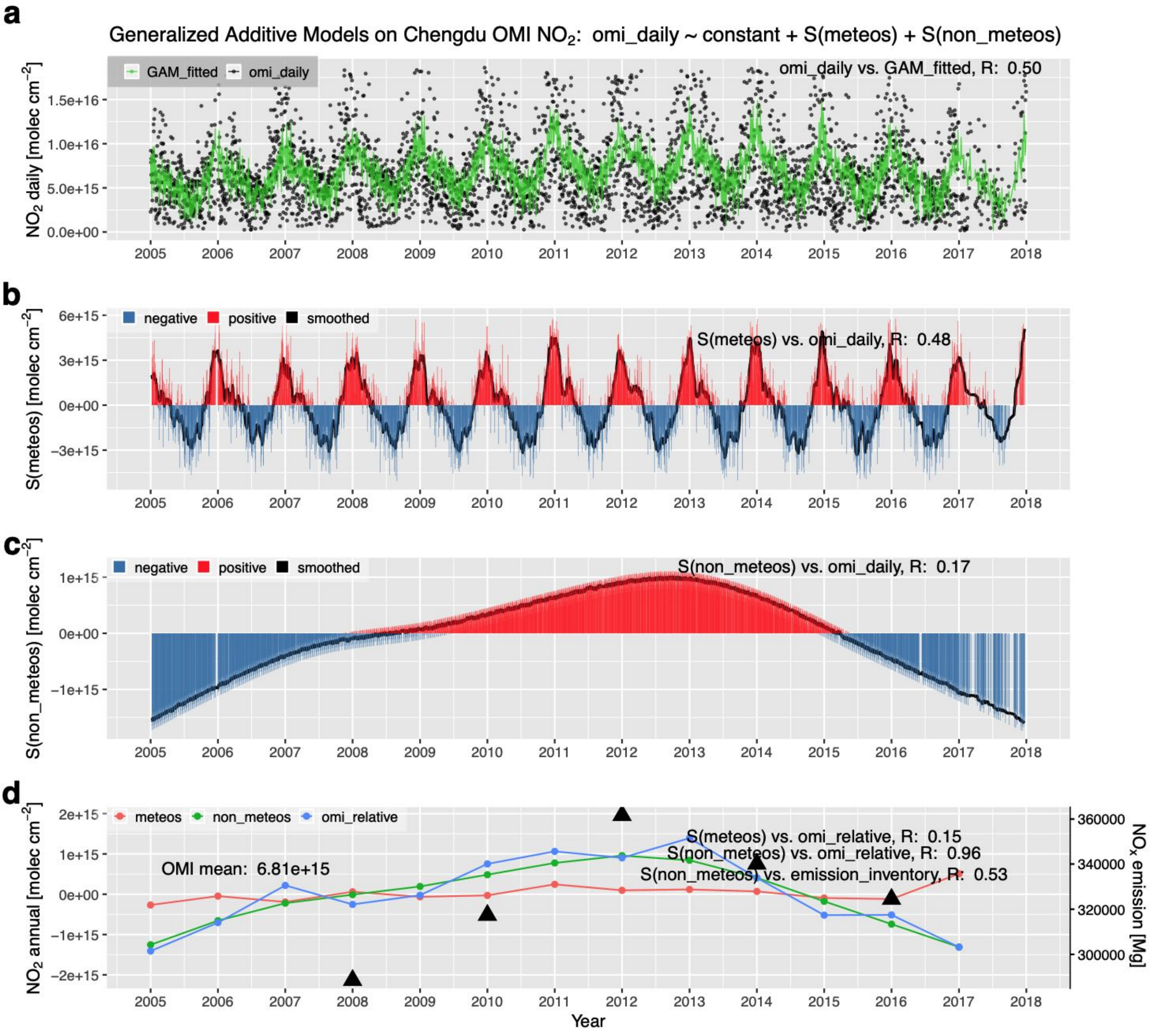

Figure S15. Similar to Fig. 3, but for NO2 in Chengdu

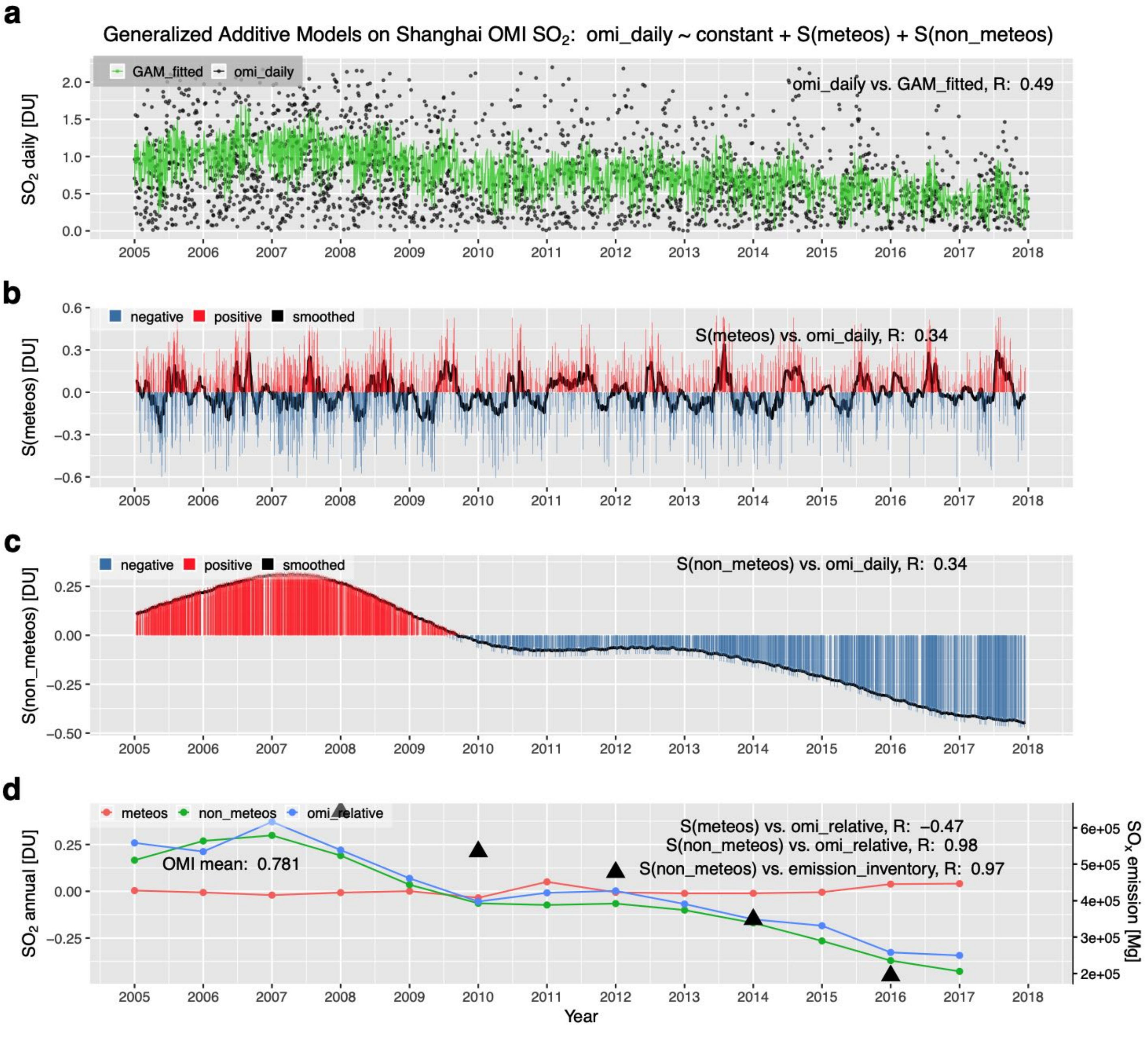

Figure S16. Similar to Fig. 4, but for SO_2 in Shanghai

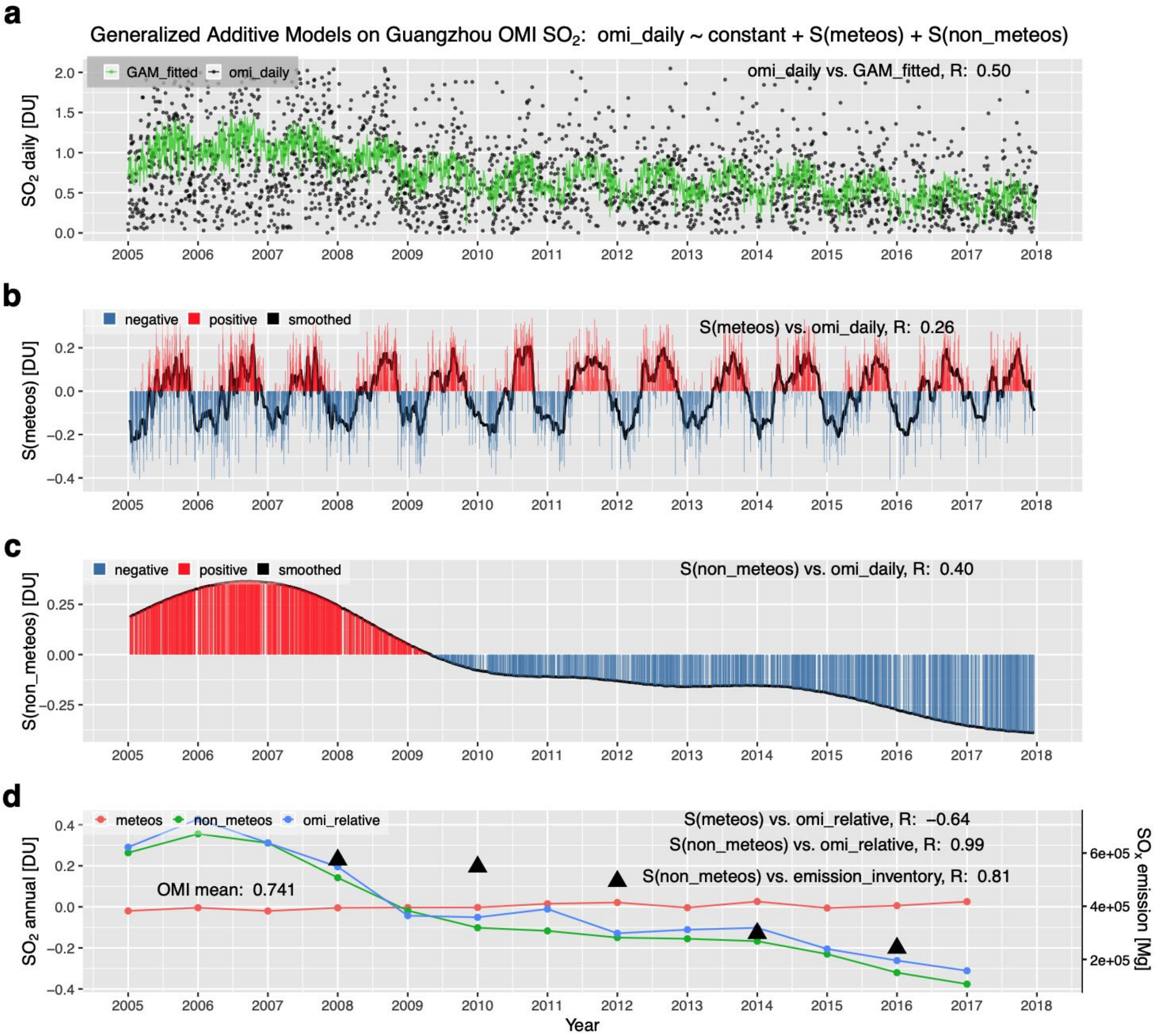

Figure S17. Similar to Fig. 4, but for SO_2 in Guangzhou

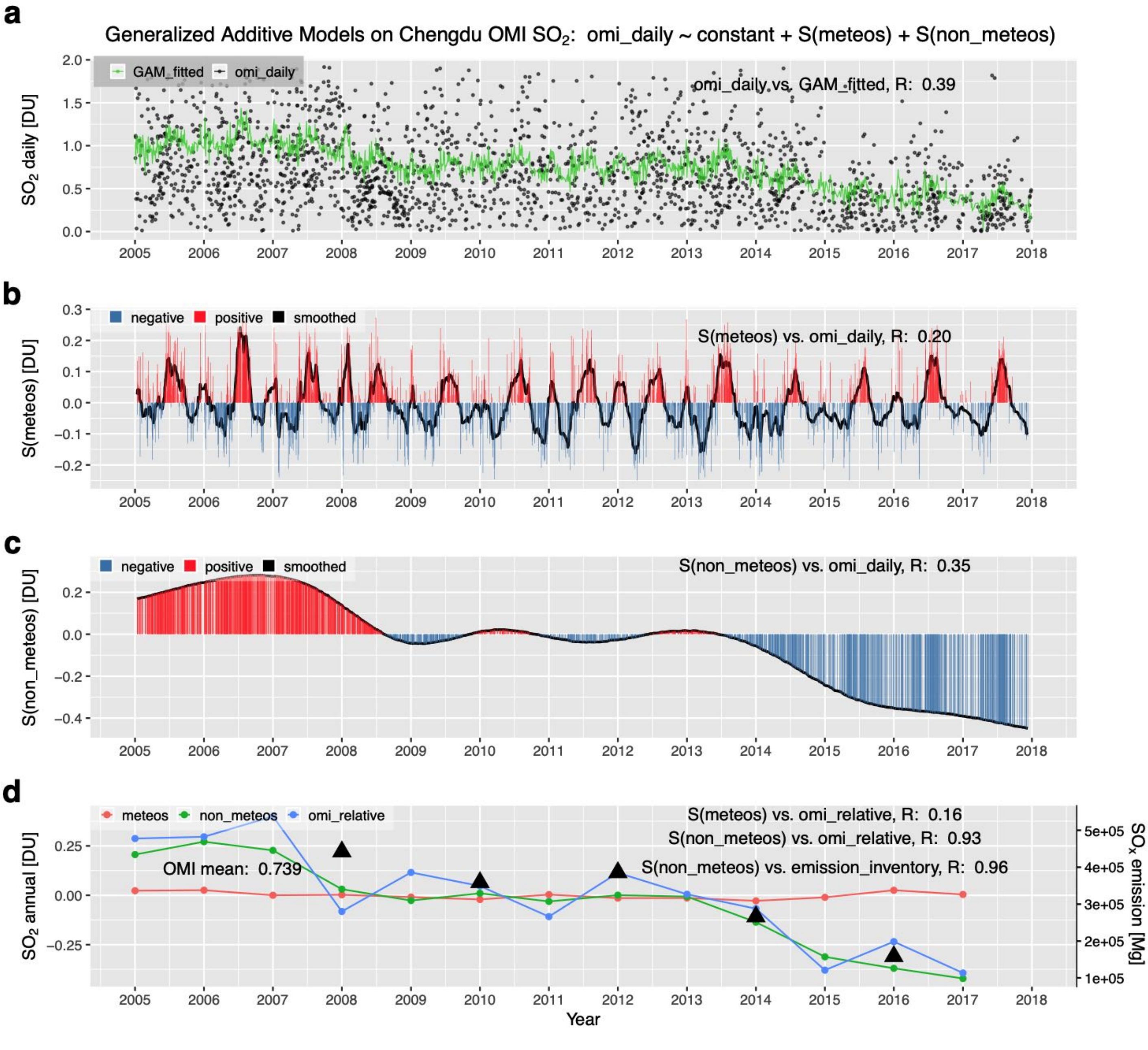

Figure S18. Similar to Fig. 4, but for SO_2 in Chengdu

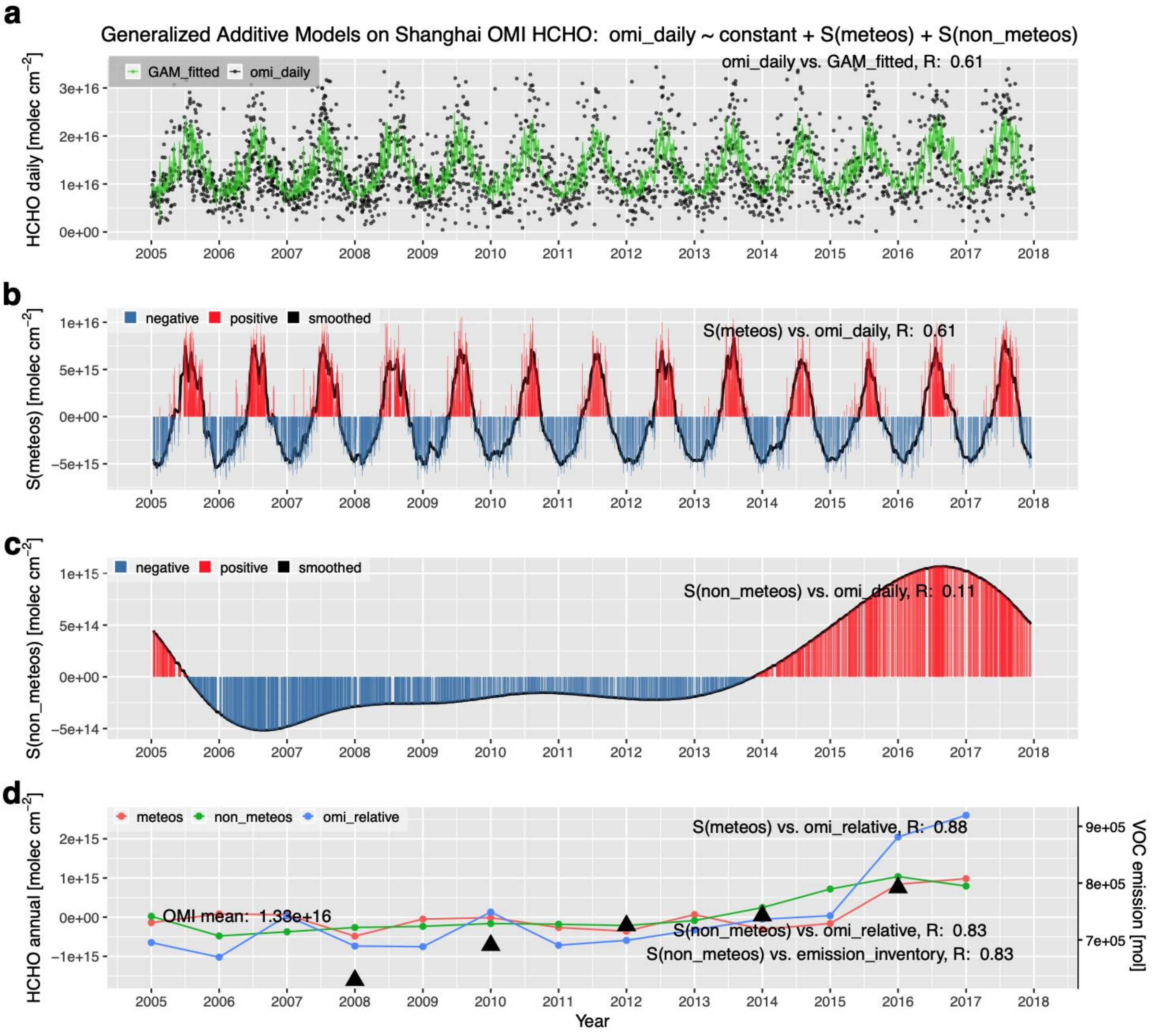

Figure S19. Similar to Fig. 5, but for HCHO in Shanghai

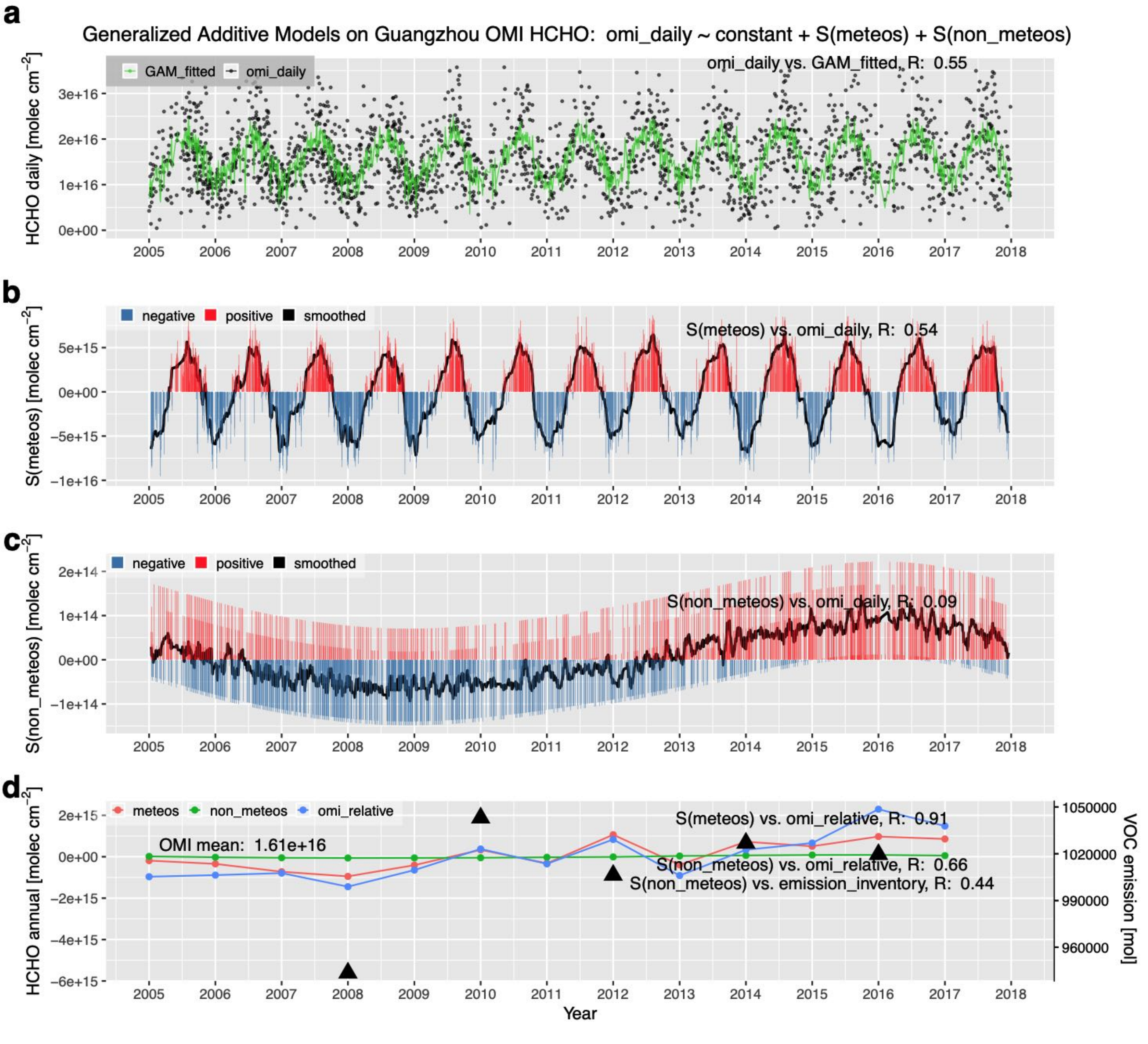

Figure S20. Similar to Fig. 5, but for HCHO in Guangzhou

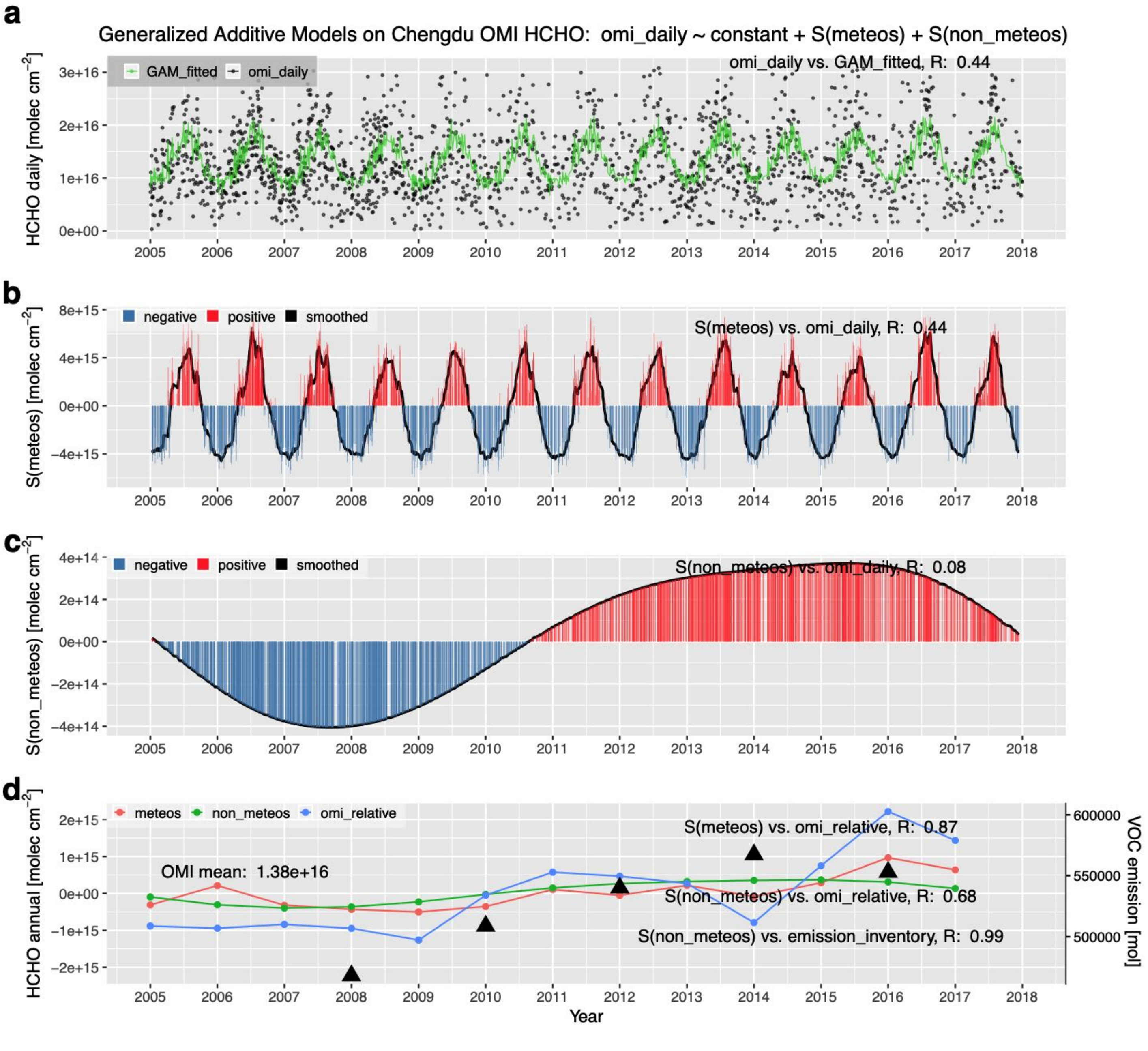

Figure S21. Similar to Fig. 5, but for HCHO in Chengdu

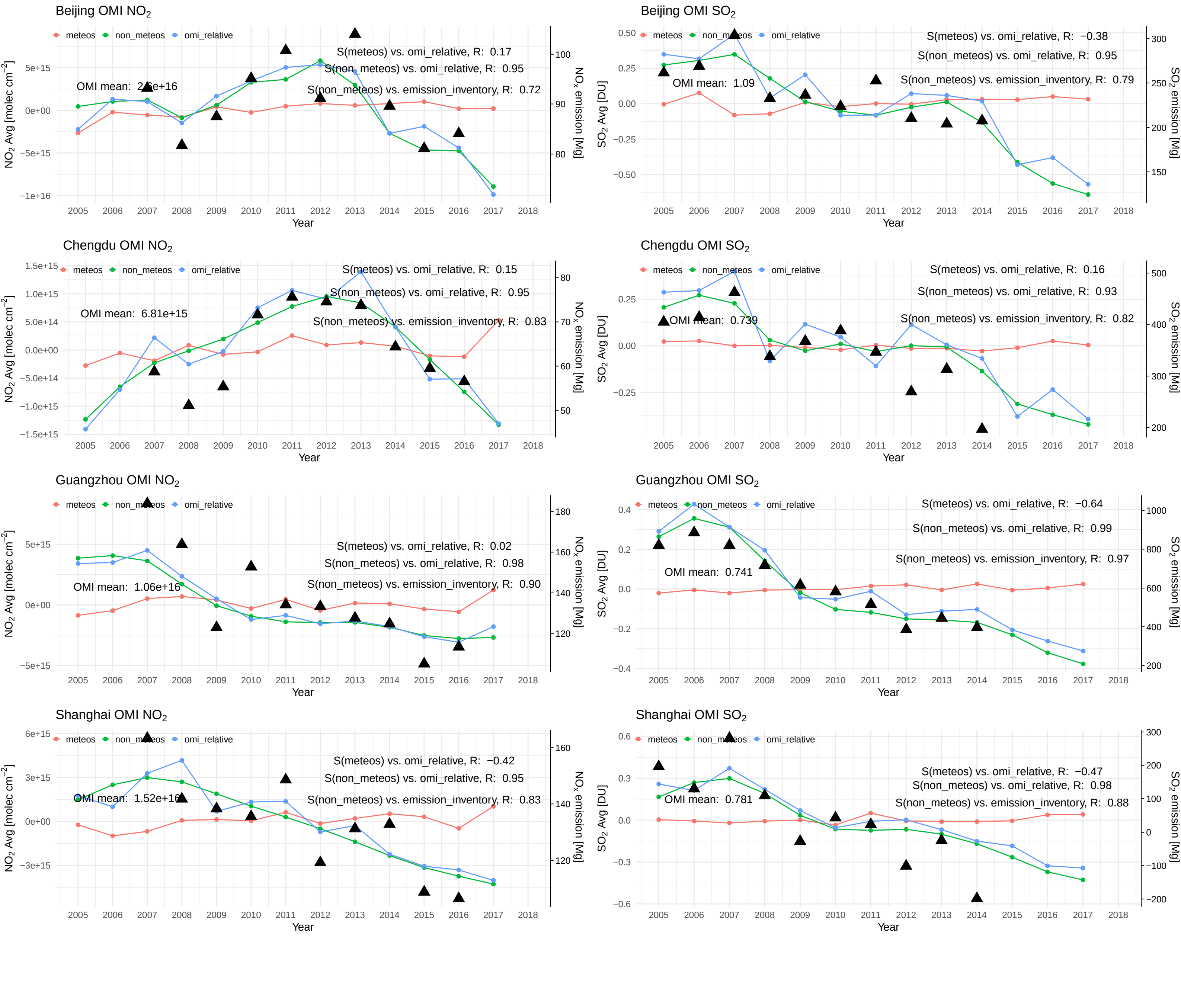

Figure S22. Similar to Figure 3d, but are additional comparisons with top-down satellite NO_x and SO_2 emission data (available at http://www.marcopolo-panda.eu/products/toolbox/emission-data/).

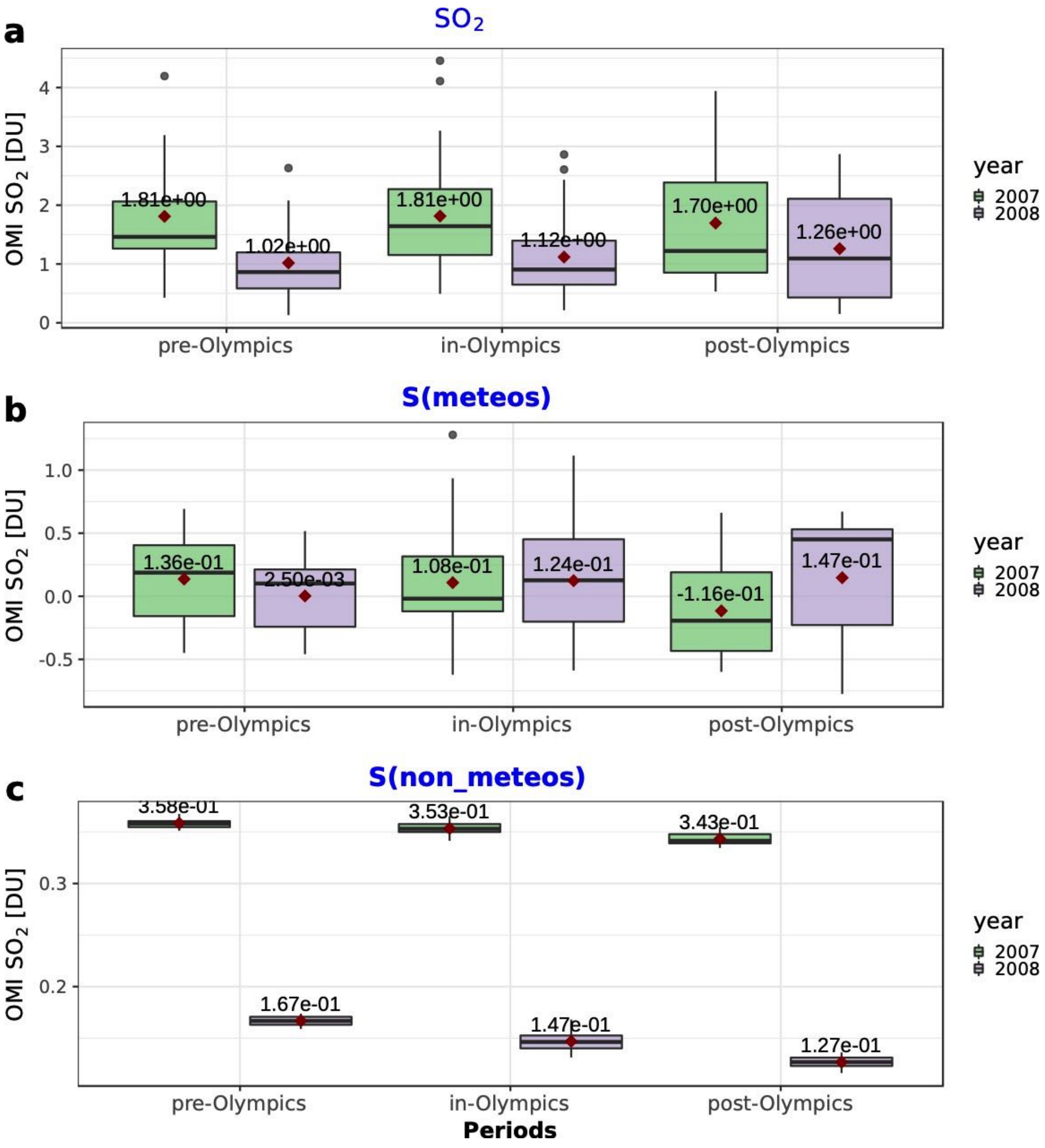

Figure S23. Similar to Fig. 6, but for SO_2 .

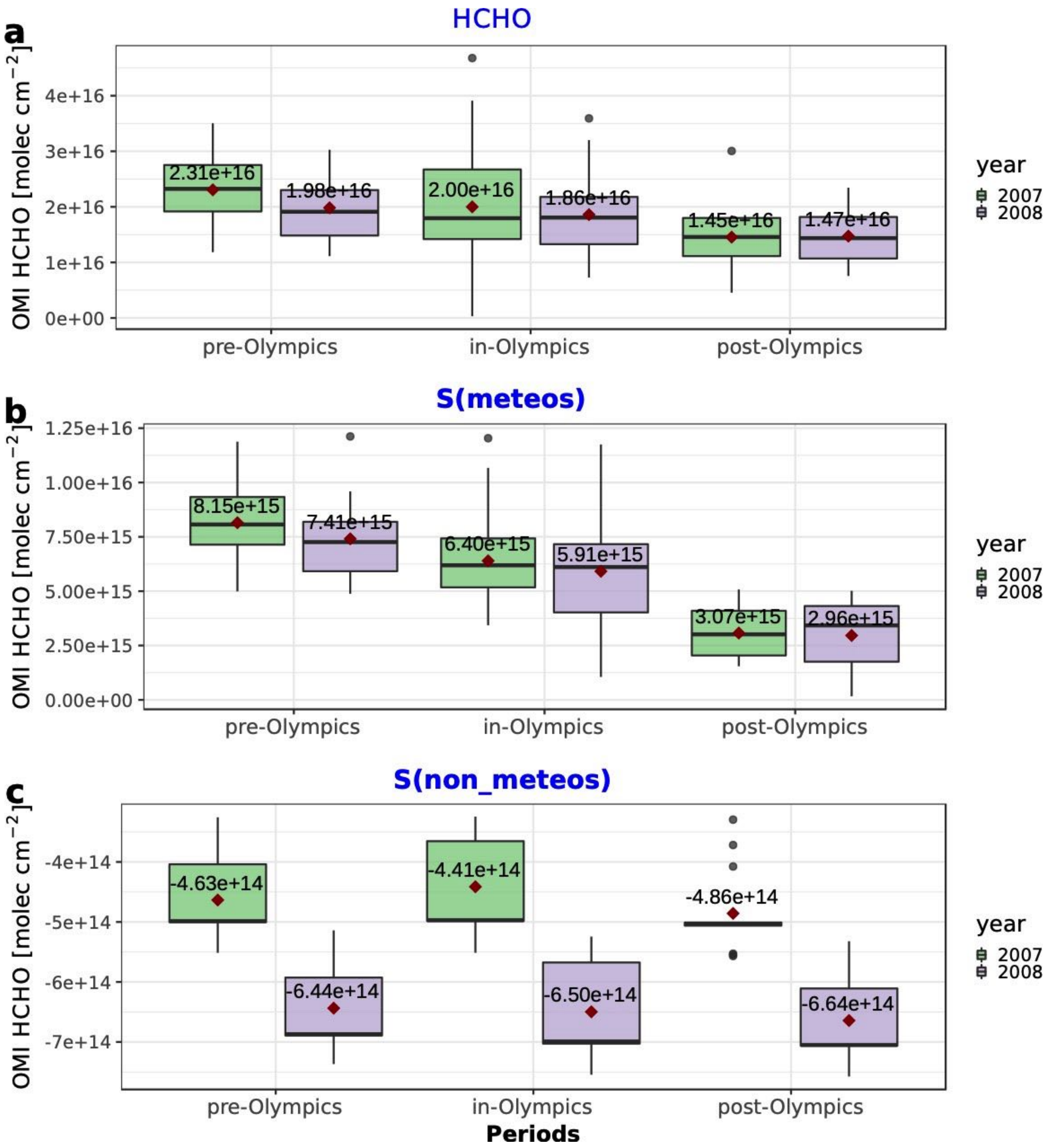

Figure S24. Similar to Fig. 6, but for HCHO

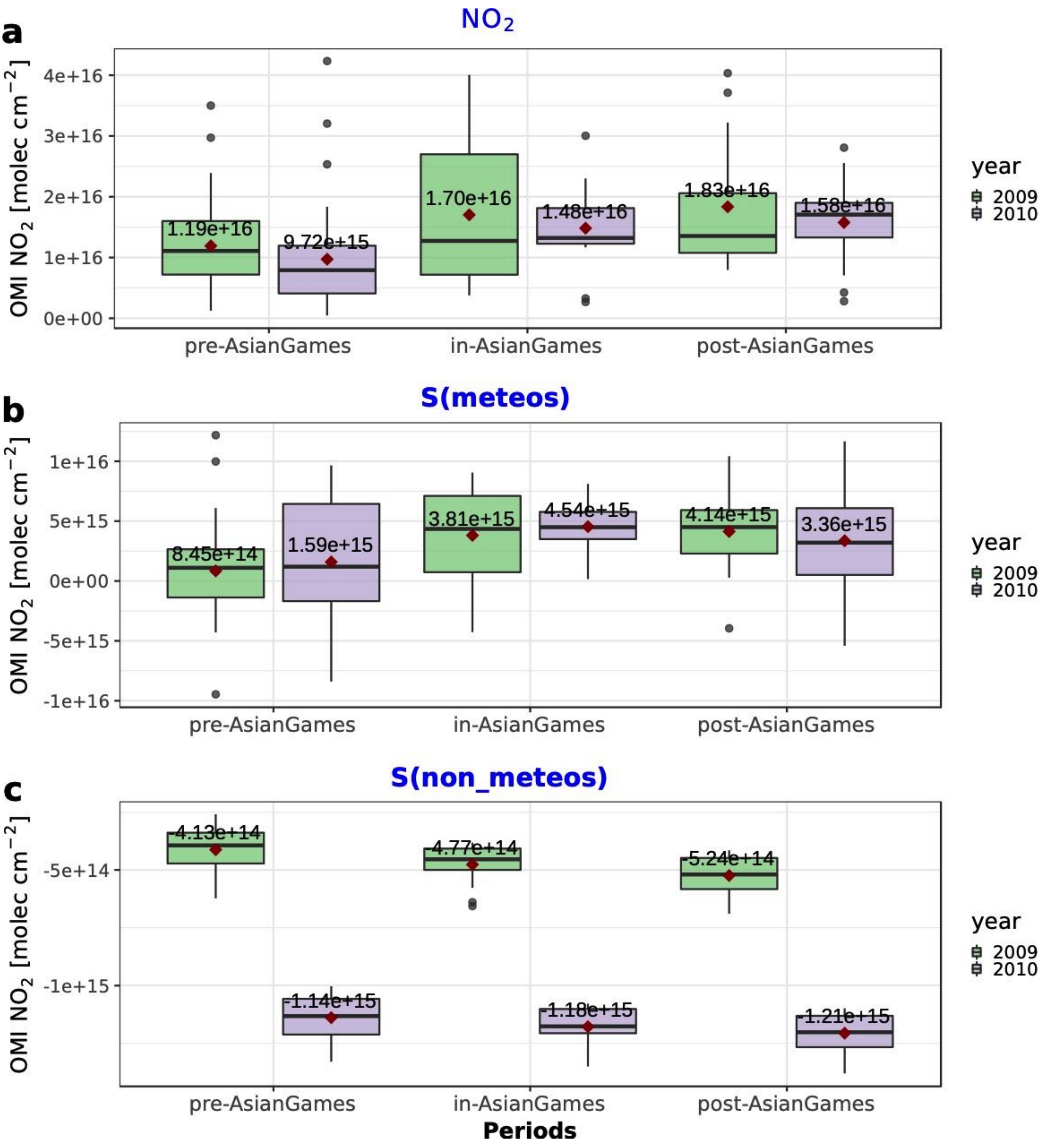

Figure S25. Similar to Fig. 6, but for NO_2 during 2010 Guangzhou Asian Games

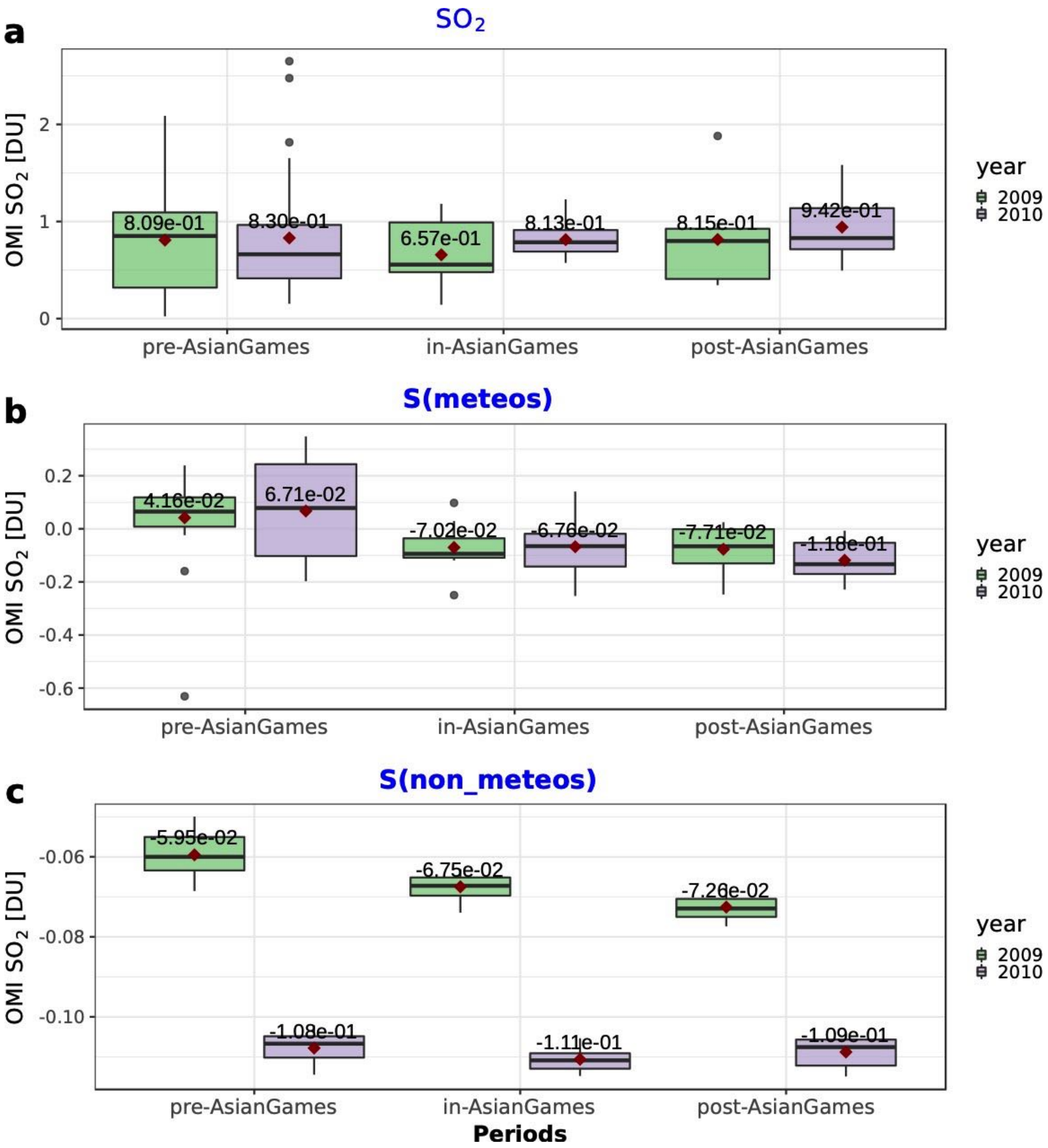

Figure S26. Similar to Fig. 6, but for SO_2 during 2010 Guangzhou Asian Games

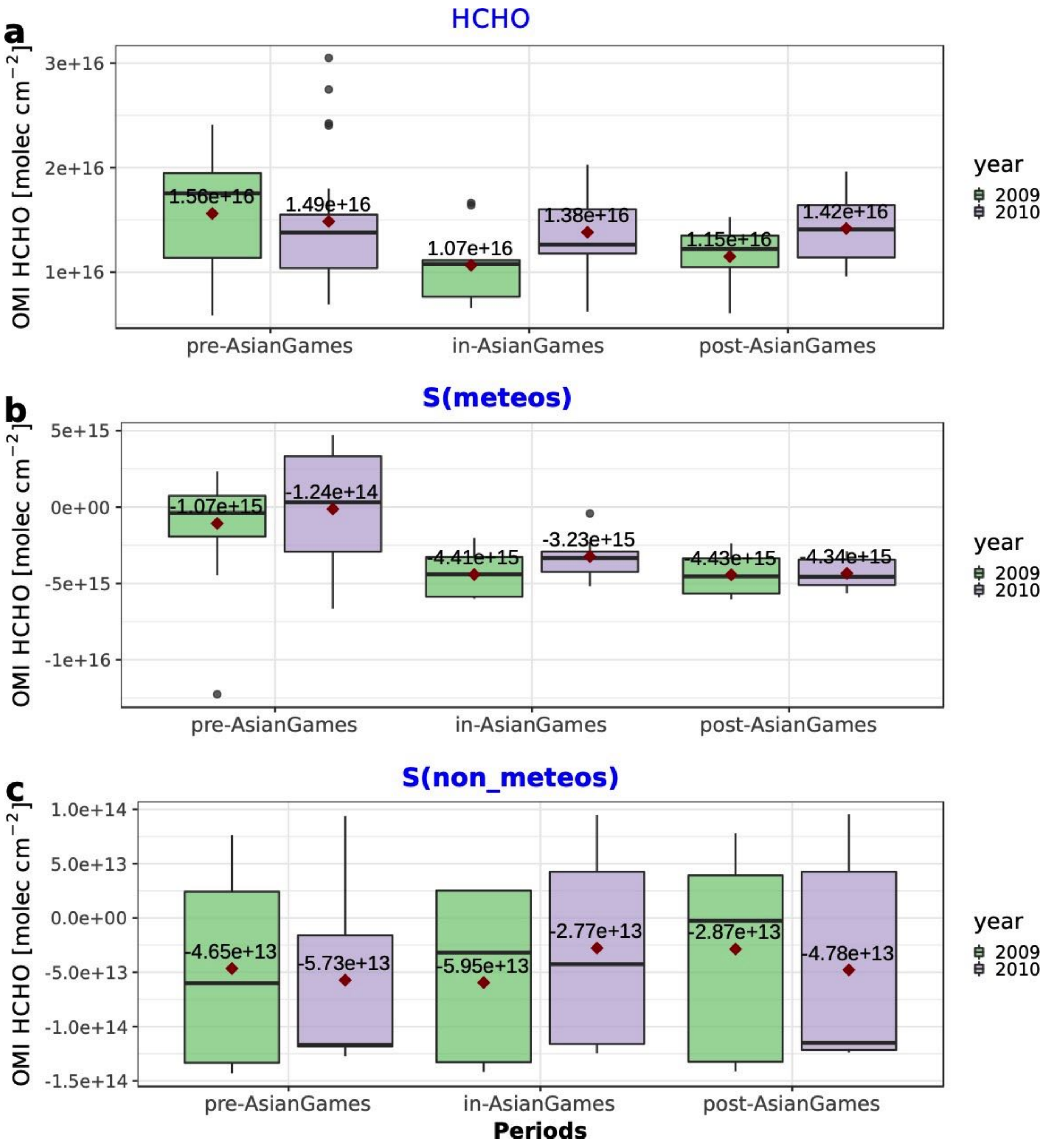

Figure S27. Similar to Fig. 6, but for HCHO during 2010 Guangzhou Asian Games
